# Catalytic and structural insights into a stereospecific and thermostable Class II aldolase HpaI from *Acinetobacter baumannii*

**DOI:** 10.1016/j.jbc.2021.101280

**Published:** 2021-10-05

**Authors:** Pratchaya Watthaisong, Asweena Binlaeh, Aritsara Jaruwat, Narin Lawan, Jirawat Tantipisit, Juthamas Jaroensuk, Litavadee Chuaboon, Jittima Phonbuppha, Ruchanok Tinikul, Pimchai Chaiyen, Penchit Chitnumsub, Somchart Maenpuen

**Affiliations:** 1 School of Biomolecular Science and Engineering, Vidyasirimedhi Institute of Science and Technology (VISTEC), Rayong, Thailand; 2 Biomolecular Analysis and Application Research Team, National Center for Genetic Engineering and Biotechnology (BIOTEC), National Science and Technology Development Agency, Pathumthani, Thailand; 3 Department of Chemistry, Faculty of Science, Chiang Mai University, Chiang Mai, Thailand; 4 Department of Biochemistry, Faculty of Science, Burapha University, Chonburi, Thailand; 5 School of Pharmacy, Walailak University, Nakhon Si Thammarat, Thailand; 6 Department of Biochemistry and Center for Excellence in Protein and Enzyme Technology, Faculty of Science, Mahidol University, Bangkok, Thailand

**Keywords:** pyruvate-specific Class II metal aldolase, metal-dependent enzyme, enzyme catalysis, crystal structure, structure–function, thermostable enzyme, solvent-tolerant enzyme, stereospecificity, stereoselectivity, *p*-hydroxyphenylacetate degradation pathway, (4*R*)-KDGal, (4*R*)-2-keto-3-deoxy-D-galactonate, (4*S*)-KDGlu, (4*S*)-2-keto-3-deoxy-D-gluconate, *Ab*HpaI, 4-hydroxy-2-ketoheptane-1,7-dioate aldolase from *Acinetobacter baumannii*, BSA, bovine serum albumin, DHAP, dihydroxyacetone phosphate, DTT, dithiothreitol, *Ec*HpaI, 4-hydroxy-2-ketoheptane-1,7-dioate aldolase from *Escherichia coli*, EDTA, ethylenediaminetetraacetatic acid, EGTA, ethylene glycol-bis(2-aminoethylether)-*N,N,N′,N′*-tetraacetic acid, FPLC, fast protein liquid chromatography, HBA, 4-hydroxybenzaldehyde, HEPES, 4-(2-hydroxyethyl)-1-piperazineethanesulfonic acid, HKHD, 4-hydroxy-2-ketoheptane-1,7-dioate, HOPA, 4-hydroxy-2-oxopentanoate, HNO_3_, nitric acid, ICP-OES, inductively coupled plasma-optical emission spectrometry, LC-ESI-QTOF-MS, liquid chromatography-electrospray ionization-quadrupole-time-of-flight mass spectrometer, LDH, lactate dehydrogenase, M^2+^, divalent meatl ion, MD, molecular dynamics, MPD, 2-methyl-2,4-pentanediol, MW, molecular weight, NaCl, sodium chloride, NADH, the reduced β-nicotinamide adenine dinucleotide, (NH_4_)_2_SO_4_, ammonium sulfate, OAA, oxaloacetate, PDB, Protein Data Bank, PEI, polyethyleneimine, PMSF, phenyl methane sulfonyl fluoride, PPA, propionaldehyde, PYR, pyruvate, QM/MM, quantum mechanics/molecular mechanics, SEC, size-exclusion chromatography, SSA, succinic semialdehyde, *T*_*m*_, melting temperature

## Abstract

Aldolases catalyze the reversible reactions of aldol condensation and cleavage and have strong potential for the synthesis of chiral compounds, widely used in pharmaceuticals. Here, we investigated a new Class II metal aldolase from the *p*-hydroxyphenylacetate degradation pathway in *Acinetobacter baumannii*, 4-hydroxy-2-keto-heptane-1,7-dioate aldolase (*Ab*HpaI), which has various properties suitable for biocatalysis, including stereoselectivity/stereospecificity, broad aldehyde utilization, thermostability, and solvent tolerance. Notably, the use of Zn^2+^ by *Ab*HpaI as a native cofactor is distinct from other enzymes in this class. *Ab*HpaI can also use other metal ion (M^2+^) cofactors, except Ca^2+^, for catalysis. We found that Zn^2+^ yielded the highest enzyme complex thermostability (*T*_*m*_ of 87 °C) and solvent tolerance. All *Ab*HpaI•M^2+^ complexes demonstrated preferential cleavage of (4*R*)-2-keto-3-deoxy-D-galactonate ((4*R*)-KDGal) over (4*S*)-2-keto-3-deoxy-D-gluconate ((4*S*)-KDGlu), with *Ab*HpaI•Zn^2+^ displaying the highest *R*/*S* stereoselectivity ratio (sixfold higher than other M^2+^ cofactors). For the aldol condensation reaction, *Ab*HpaI•M^2+^ only specifically forms (4*R*)-KDGal and not (4*S*)-KDGlu and preferentially catalyzes condensation rather than cleavage by ∼40-fold. Based on 11 X-ray structures of *Ab*HpaI complexed with M^2+^ and ligands at 1.85 to 2.0 Å resolution, the data clearly indicate that the M^2+^ cofactors form an octahedral geometry with Glu151 and Asp177, pyruvate, and water molecules. Moreover, Arg72 in the Zn^2+^-bound form governs the stereoselectivity/stereospecificity of *Ab*HpaI. X-ray structures also show that Ca^2+^ binds at the trimer interface *via* interaction with Asp51. Hence, we conclude that *Ab*HpaI•Zn^2+^ is distinctive from its homologues in substrate stereospecificity, preference for aldol formation over cleavage, and protein robustness, and is attractive for biocatalytic applications.

Aldolases catalyze reversible reactions of carbon–carbon bond formation (aldol condensation) and breakage (aldol cleavage). Based on their different catalytic mechanisms, aldolases can be classified into three groups, including pyridoxal 5′-phosphate (PLP)-dependent, Class I lysine-dependent, and Class II metal-dependent aldolases. PLP aldolases employ PLP as a cofactor to react with an amino-containing nucleophilic substrate to form a quasi-stable carbanion and an iminium intermediate. Class I lysine aldolases (also called Schiff base-forming aldolases) utilize an active lysine residue to form a Schiff base with an aldehyde/keto substrate to also result in an iminium intermediate. This imine intermediate is susceptible to C-C bond cleavage or formation. For Class II metal aldolases, the enzyme uses a divalent metal ion (M^2+^) as a cofactor for substrate binding and stabilization of an enolate intermediate (1, 2, 3, 4, 5, 6), which allows the reaction to proceed through C-C bond formation or cleavage.

These aldolases are capable of catalyzing stereochemically-specific reactions, offering attractive and interesting routes for synthesis of rare sugars, β- and γ-hydroxy-α-amino acids, optically pure compounds, and antiviral agents to be used in pharmaceuticals (1, 2, 3, 6, 7, 8, 9, 10, 11, 12, 13). For PLP aldolases, two known enzymes—serine hydroxymethyltransferase (SHMT) and threonine aldolase (TA), which are capable of synthesizing nonnatural β-hydroxy-α-amino acids such as β-hydroxy-α,α-dialkyl-α-amino acids or L-*threo*-3,4-dihydroxyphenylserine and β-phenylserine (1, 5, 6, 14), have been studied. A wide range of Class I lysine aldolases have been investigated due to their diversified reactions. For example, 2-deoxyribose 5-phosphate aldolase (DERA), *N*-acetylneuraminic acid aldolase (NeuA), and D-fructose 1,6-bisphosphate aldolase (FruA) have been extensively used in industrial applications to synthesize active pharmaceutical ingredients (APIs) (15, 16, 17, 18). In contrast, applications of Class II metal aldolases have been much less investigated, but have recently been gaining more interest for their applications in the stereoselective synthesis of rare sugars. For example, the rare sugars L-fructose, D-sorbose, and D-psicose can be synthesized from the reaction of rhamnulose 1-phosphate aldolase (RhuA or RhaD) (18). As Class II metal aldolases are generally more thermostable than the Class I enzymes (2, 19), these enzymes thus receive increasing attention in biocatalysis due to their robustness (4, 14, 20, 21, 22, 23, 24, 25).

Reactions and properties of Class II metal aldolases are diversified as the enzymes can use a wide range of M^2+^ and carbonyl group substrates. Pyruvate-specific aldolases such as 4-hydroxy-2-keto-heptane-1,7-dioate aldolase (HpaI), 4-hydroxy-2-ketovalerate aldolase (BphI and DmpG) and 2-keto-3-deoxy-L-rhamnonate aldolase (YfaU) can use various octahedrally coordinated Mg^2+^, Co^2+^, or Mn^2+^ ions as cofactors (23, 24, 26, 27, 28, 29, 30, 31, 32, 33), while dihydroxyacetone phosphate (DHAP)-specific aldolases such as FruA and L-fucose 1-phosphate aldolase (FucA) generally bind and use a tetrahedrally coordinated Zn^2+^ cofactor (2, 34, 35, 36, 37). In general, differences in M^2+^ coordination geometry can affect the rate and reaction specificity of metalloenzymes (38). In the case of pyruvate-dependent aldolases, biophysical factors governing the ability of these enzymes to bind various types of M^2+^ and the mechanistic roles of these M^2+^ in catalysis are unclear.

The most well-studied pyruvate-specific Class II metal aldolase is HpaI (EC 4.1.2.52) found in the *p*-hydroxyphenylacetate (HPA) degradation pathway in *Escherichia coli* (*Ec*HpaI). The enzyme catalyzes the reversible aldol cleavage of 4-hydroxy-2-keto-heptane-1,7-dioate (HKHD) to form pyruvate and succinic semialdehyde (SSA). Crystal structures, steady-state kinetics, and substrate specificity of *Ec*HpaI indicate that the enzyme exists as a hexamer (a dimer of trimers) in which each subunit can bind to an octahedral divalent metal ion such as Mg^2+^, Mn^2+^, or Co^2+^ coordinated with substrates pyruvate (20, 23, 26, 27, 30, 39, 40). Results from quantum mechanics/molecular mechanics (QM/MM) calculations and site-directed mutagenesis studies indicate that Arg70 and His45 together with the M^2+^-bound apex water molecule are important for substrate specificity, C-C bond cleavage, and enolate stabilization (27, 30, 39, 40). *Ec*HpaI can also catalyze the aldol condensation of keto donors (pyruvate or 2-ketobutyrate) and various types of aldehyde acceptors of different carbon chain lengths (C_2_–C_5_) to generate the corresponding 4-hydroxy-2-ketoacids with preference toward a longer chain C_5_-aldehyde (pentaldehyde) rather than other short-chain aldehydes (23). Although *Ec*HpaI can use a broad range of aldehydes, its reaction lacks stereospecificity (23). Therefore, a new aldolase with similar catalytic capability as *Ec*HpaI but capable of catalyzing stereospecific reactions with thermostability would be a more preferred biocatalyst.

Our group has identified a new HpaI from the HPA degradation pathway in *Acinetobacter baumannii* (*Ab*HpaI), which shares 59% amino acid sequence identity with *Ec*HpaI (Fig. S1) (41). Structures and catalytic properties of *Ab*HpaI have never been investigated. In this work, we investigated the catalytic and biophysical properties of *Ab*HpaI and found that the enzyme has biochemical and biophysical properties significantly different from *Ec*HpaI and other enzymes in this class such as the use of Zn^2+^ as a cofactor. We also showed that *Ab*HpaI can catalyze stereospecific aldol condensation to synthesize pure (4*R*)-2-keto-3-deoxy-D-galactonate ((4*R*)-KDGal) without producing the contaminating 4*S*-isomer, demonstrating that *Ab*HpaI can control the stereospecificity of aldol product formation. Steady-state kinetics indicate that the turnover number of aldol condensation to synthesize (4*R*)-KDGal was about 35- to 40-fold faster than that the cleavage, suggesting that the aldol condensation is a more favored direction of *Ab*HpaI catalysis. Moreover, *Ab*HpaI is also tolerant to various solvents and highly thermostable especially in the Zn^2+^-bound form in which *T*_*m*_ is 87 °C. We solved 11 X-ray structures of *Ab*HpaI in complex with various M^2+^ and substrates to elucidate the structural factors underlying the catalysis of *Ab*HpaI. Structual analysis clearly explains why the 4*R*-isomer is more preferred over the 4*S*-isomer for cleavage and how different M^2+^ cofactors affect the binding features of both substrates. Arg72 is the key residue governing the stereochemistry of *Ab*HpaI. Together, these properties, which are quite different from *Ec*HpaI, make *Ab*HpaI attractive as a robust biocatalyst for aldol condensation to produce the stereospecific/stereoselective 4-hydroxy-2-ketoacid synthons for further preparation of APIs.

## Results

### Identification of the native metal ion cofactor for AbHpaI

**Figure 1:**
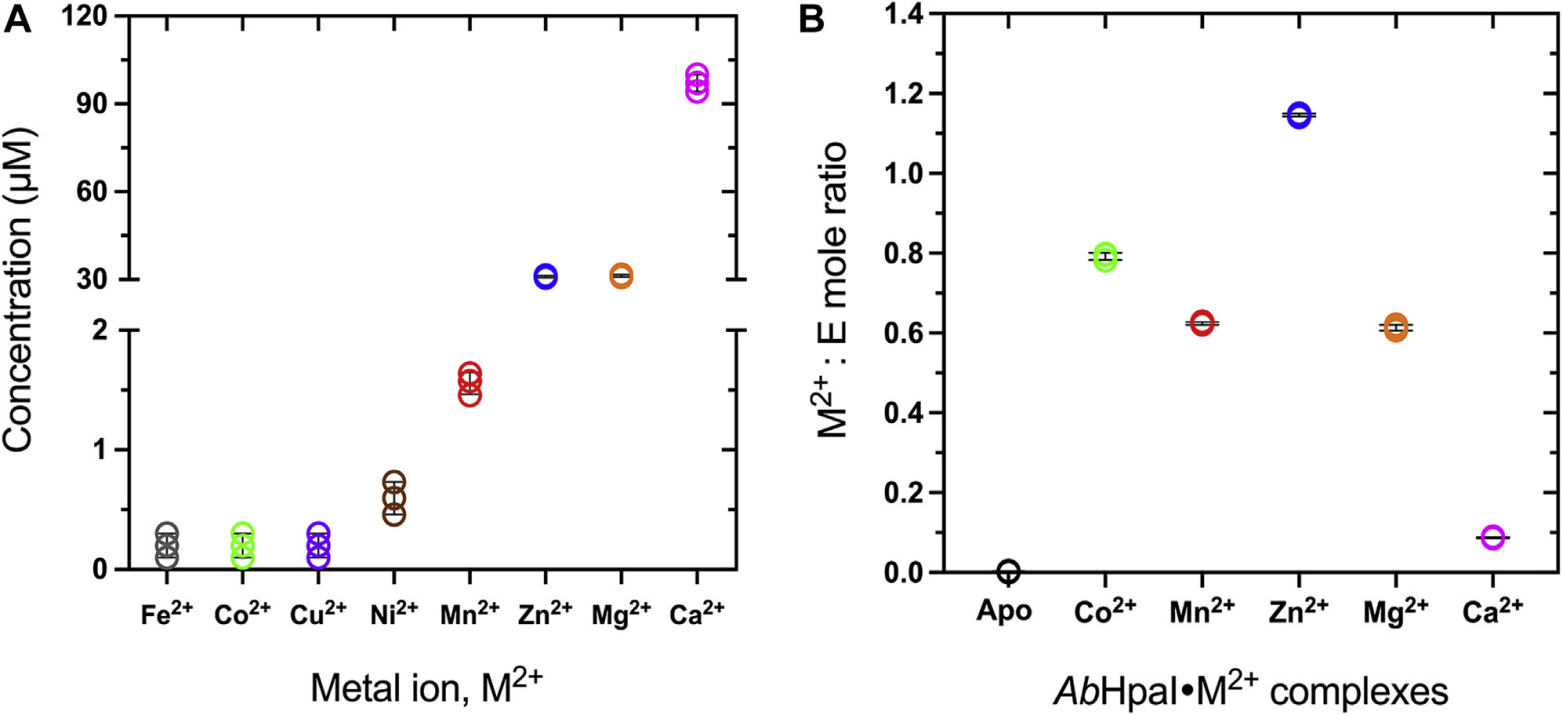
**Identification of metal ions bound to *Ab*HpaI by ICP-OES analysis.***A*, the metal ion contents found in the purified *Ab*HpaI (270 μM). *B*, the mole ratio of metal ion per *Ab*HpaI in the reconstituted *Ab*HpaI•M^2+^ complex. *Error bars* represent standard deviations (S.D.) from three replications of the data.

We first explored the selectivity of metal ion binding in *Ab*HpaI and identified its native cofactor. Using inductively coupled plasma–optical emission spectroscopy (ICP-OES), which can detect a wide variety of metal ions simultaneously, alkaline earth ions (Ca^2+^ and Mg^2+^) and transition metal ions (Zn^2+^, Mn^2+^, and Ni^2+^) were detected in the purified *Ab*HpaI (Fig. 1*A*). Quantitative measurements indicated that Ca^2+^ ion was the most prevalent, followed by Zn^2+^ and Mg^2+^, while Mn^2+^ and Ni^2+^ were found in very low amounts (Fig. 1*A*). In contrast to the properties of *Ec*HpaI, which could bind to three metal ions (Mn^2+^, Mg^2+^, Co^2+^) with Co^2+^ giving the highest activity (26), Co^2+^ was not found in the purified *Ab*HpaI.

As the presence of metal ions in the purified *Ab*HpaI may not directly relate to the enzyme catalytic activity because their existence may depend on their availability in cells, we thus further investigated the effects of different metal ions on the catalysis of *Ab*HpaI. First, the binding properties of these metal ions to apo-*Ab*HpaI and in the presence of pyruvate substrate were determined. Apo-*Ab*HpaI was reconstituted with each metal ion, namely Zn^2+^, Mn^2+^, Co^2+^, Ca^2+^, and Mg^2+^ and ability of these M^2+^ to bind to the apoenzyme was determined. Although Co^2+^ was not found in the purified *Ab*HpaI (Fig. 1*A*), we included Co^2+^ in this study because it is a native cofactor of *Ec*HpaI (26, 27, 39). The ICP-OES results (Fig. 1*B*) showed that the mole ratios of each of the reconstituted metal ions Zn^2+^, Mn^2+^, Co^2+^, Mg^2+^, and Ca^2+^ to apo-*Ab*HpaI varied from 1.1, 0.6, 0.8, 0.6, and 0.1, respectively, suggesting that *Ab*HpaI has different affinities and preferences toward these metal ions. Unexpectedly, Ca^2+^ has the lowest binding ability to *Ab*HpaI after reconstitution, albeit Ca^2+^ was the most detected ion in the purified enzyme. We further investigated the binding constant (*K*_d_) of M^2+^ and pyruvate in *Ab*HpaI•M^2+^ and *Ab*HpaI•M^2+^•pyruvate complexes using isothermal titration calorimetry (ITC). *Ab*HpaI has three and sevenfold greater affinity for Zn^2+^ binding over Co^2+^ and Mn^2+^, respectively, while the *K*_d_ of Mg^2+^ and Ca^2+^ to *Ab*HpaI could not be determined (Table 1). Together with the finding that Zn^2+^ has the highest mole ratios in metal ion reconstitution experiments and in the native purified enzyme, these results suggest that Zn^2+^ is the native cofactor for *Ab*HpaI.

**Table 1:** Thermodynamic and catalytic properties of AbHpaI reconstituted with different metal ion cofactors

*Ab*HpaI	*K*_d, metal ion_ (μM)	*K*_d, pyruvate_ (μM)	*T*_*m*_ (°C)	Δ*T*_*m*_^a^
Apo-*Ab*HpaI	-	ND	81.3 ± 0.6	0.0
*Ab*HpaI•Zn^2+^	3.7 ± 2.1	980 ± 60	87.0 ± 0.5	5.7
*Ab*HpaI•Co^2+^	11.4 ± 0.1	320 ± 40	84.7 ± 0.6	3.4
*Ab*HpaI•Mn^2+^	25.5 ± 4.8	1320 ± 500	83.0 ± 1.0	1.7
*Ab*HpaI•Mg^2+^	ND	ND	81.7 ± 1.1	0.4
*Ab*HpaI•Ca^2+^	ND	ND	81.3 ± 0.5	0.0

Abbreviation: ND, not detectable.

a The Δ*T*_*m*_ values were calculated by subtracting the *T*_*m*_ value of the apo-*Ab*HpaI from that of the metal ion-bound *Ab*HpaI.

As pyruvate alone cannot bind to apo-*Ab*HpaI, we thus further explored the role of the five metal ions in facilitating the binding of pyruvate to *Ab*HpaI by measuring the binding constant of pyruvate using ITC. We found that only the transition metal ions Zn^2+^, Co^2+^, and Mn^2+^ could support the binding of pyruvate in which *Ab*HpaI•Co^2+^ has a three and fourfold higher affinity to pyruvate than *Ab*HpaI•Zn^2+^ and *Ab*HpaI•Mn^2+^ (Table 1). Notably, the *K*_d_ values of pyruvate binding to *Ab*HpaI•Mg^2+^ and *Ab*HpaI•Ca^2+^ could not be measured, indicating that pyruvate has poor affinity to these enzyme complexes. However, apparent kinetic results showed that *Ab*HpaI•Mg^2+^ could catalyze the aldol condensation reaction of pyruvate and D-glyceraldehyde with 1.3-fold slower than *Ab*HpaI•Zn^2+^, while *Ab*HpaI•Ca^2+^ could not (Table 2). These suggest that Zn^2+^, Co^2+^, Mn^2+^, and Mg^2+^ but not Ca^2+^ have properties relevant to being metal ion cofactors. In addition, our work here indicates that an enzyme in the pyruvate-specific Class II metal aldolases can use Zn^2+^ as a catalytic cofactor.

**Table 2:** Stereoselectivity of substrates in the aldol cleavage and stereochemistry of product formation in the condensation of *Ab*HpaI with different metal ion cofactors

*Ab*HpaI	Aldol cleavage activity^a^ (μM/min)		*R*/*S* ratio	Aldol condensation activity^b^ (μM/min)	
	(4*R*)-KDGal	(4*S*)-KDGlu		(4*R*)-KDGal	(4*S*)-KDGlu
Apo-*Ab*HpaI	none^c^	none^c^	-	none^c^	none^c^
*Ab*HpaI•Zn^2+^	82.9 ± 4.4	0.8 ± 0.1	104	14.0 ± 0.3	
*Ab*HpaI•Co^2+^	171.1 ± 6.5	10.8 ± 1.7	16	26.6 ± 1.0	
*Ab*HpaI•Mn^2+^	87.8 ± 11.4	7.8 ± 0.8	11	13.2 ± 0.4	
*Ab*HpaI•Mg^2+^	29.9 ± 3.8	1.6 ± 0.1	19	10.7 ± 0.5	
*Ab*HpaI•Ca^2+^	none^c^	none^c^	-	none^c^	

a Detection of products from the aldol cleavage reactions was carried out by coupling with the reaction of lactate dehydrogenase (LDH) in buffer H containing 0.2 mM substrate ((4*R*)-KDGal or (4*S*)-KDGlu), 0.2 mM NADH, 0.5 mM metal ion, 26.2 μg/ml LDH, and 100 μM metal ion-reconstituted *Ab*HpaI. The NADH absorbance decrease at 340 nm refers to the cleavage of the substrate to yield pyruvate for LDH reaction for 3 min.

b The aldol condensation reactions were carried out for 1 h in buffer H containing 4 mM pyruvate, 30 mM D-glyceraldehyde, 0.1 mM metal ion, 0.05 μM metal ion-reconstituted *Ab*HpaI. Product was analyzed by Agilent 6470 triple-quadrupole LC/MS.

c None, no reaction occurred under this condition.

### Stereochemistry of the catalytic reaction of AbHpaI

#### Stereoselectivity of the AbHpaI aldol cleavage

To investigate the influence of metal ions on the stereoselectivity of the substrate stereoisomer for aldol cleavage, *Ab*HpaI•M^2+^ complexes of Zn^2+^, Co^2+^, Mn^2+^, Mg^2+^, and Ca^2+^, prepared by equilibrating the apo-*Ab*HpaI with excess M^2+^, were employed for catalyzing the aldol cleavage of substrates, namely (4*R*)-KDGal and (4*S*)-KDGlu (see details of chemical structures in Fig. S2). (4*R*)-KDGal and (4*S*)-KDGlu were chosen as model substrates because these compounds are only different in stereo-isoforms (*R* and *S*) at the cleavage site of the C_4_-hydroxyl (C_4_-OH) group. The reaction rate of substrate cleavage was measured by coupling with the reaction of *Ab*HpaI•M^2+^ with lactate dehydrogenase (LDH) to detect NADH oxidation upon pyruvate formation.

**Figure 2:**
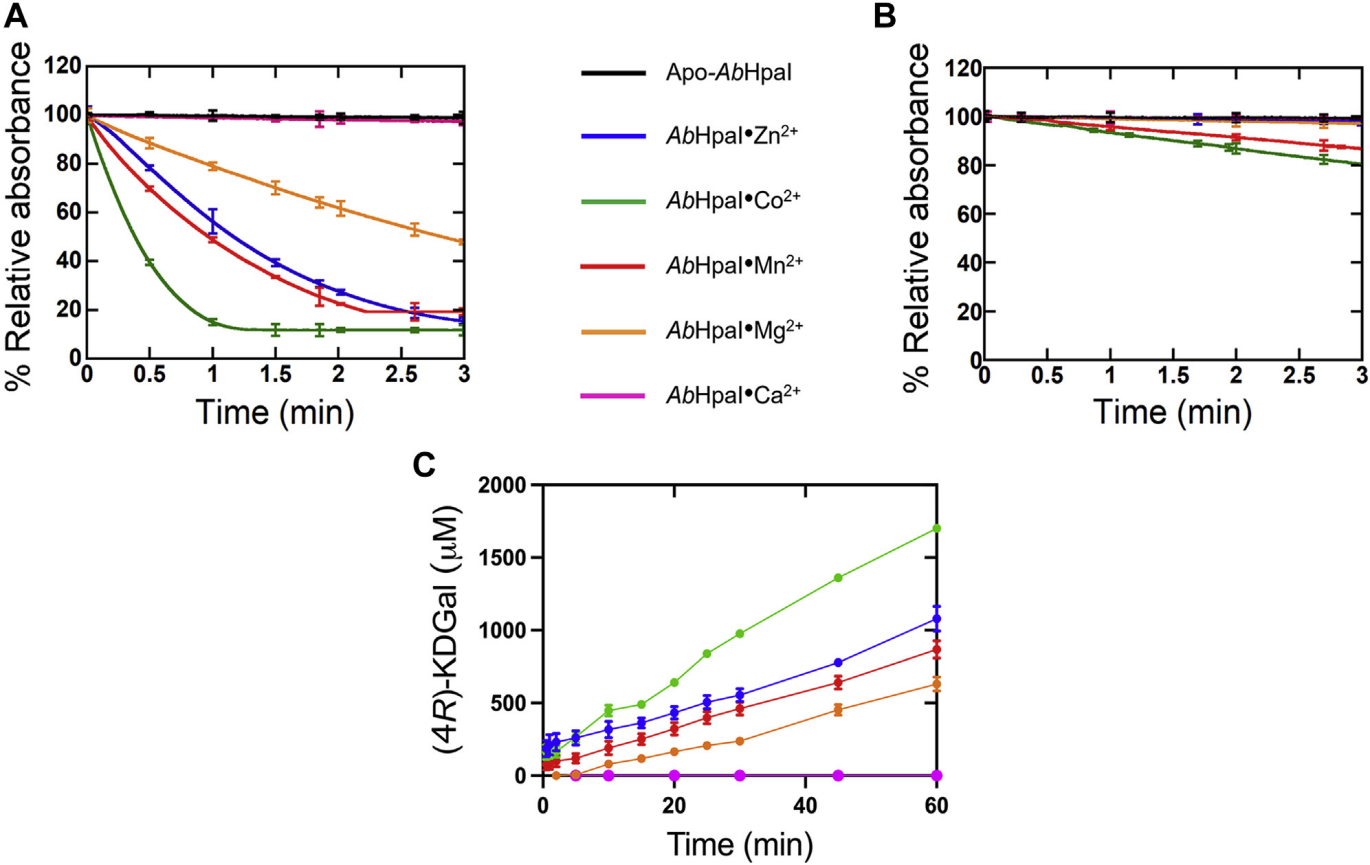
**Stereochemistry of aldol cleavage and condensation of *Ab*HpaI.** Activities of *Ab*HpaI without (*black*) and with metal ion cofactors Zn^2+^ (*blue*), Co^2+^ (*green*), Mn^2+^ (*red*), Mg^2+^ (*orange*), and Ca^2+^ (*pink*) in aldol cleavage and condensation reactions were analyzed to determine the reaction stereochemistry. In the aldol cleavage reactions, either (*A*) (4*R*)-KDGal or (*B*) (4*S*)-KDGlu was used as a substrate in the LDH-coupled assay for *Ab*HpaI activity. The relative absorbance decrease at 340 nm refers to the NADH oxidation upon conversion of pyruvate (generated from the *Ab*HpaI cleavage reaction) to form L-lactate by LDH. Therefore, the NADH oxidation can be used to represent the cleavage reaction of the 4*R*- and 4*S*-isomer substrates, depending on the substrate added. Rates of substrate cleavage by individual *Ab*HpaI•M^2+^ compelexes were determined and summarized in Table 2. *C*, products from the *Ab*HpaI•M^2+^ catalyzed aldol condensation of pyruvate and D-glyceraldehyde were analyzed by a triple-quadrupole LC/MS to detect aldol products of (4*R*)-KDGal and (4*S*)-KDGlu. All *Ab*HpaI•M^2+^ complexes, except *Ab*HpaI•Ca^2+^, can catalyze aldol condensation to form (4*R*)-KDGal. Rates of (4*R*)-KDGal formation by individual *Ab*HpaI•M^2+^ were determined and summarized in Table 2. *Error bars* represent standard deviations (S.D.) from three replications of the data.

Results in Table 2 clearly showed that the apo-*Ab*HpaI and *Ab*HpaI•Ca^2+^ cannot catalyze reactions of both isomers. For other *Ab*HpaI•M^2+^ complexes tested, they could catalyze aldol cleavage of both 4*R*- and 4*S*-isomers with higher activities toward the cleavage of (4*R*)-KDGal rather than (4*S*)-KDGlu (Table 2). The *Ab*HpaI enzymes containing Zn^2+^, Co^2+^, or Mn^2+^ cleaved 80 to 90% (4*R*)-KDGal within 3 min with *Ab*HpaI•Co^2+^ showing the fastest activity (∼90% (4*R*)-KDGal consumed within 1 min) to get pyruvate and D-glyceraldehyde, while only 50% of the (4*R*)-KDGal was utilized by *Ab*HpaI•Mg^2+^ (Fig. 2*A*). These results indicate that *Ab*HpaI with all metal ions has stereoselective preference toward (4*R*)-KDGal over (4*S*)-KDGlu.

Regarding the overall yield of (4*S*)-KDGlu cleavage (Fig. 2*B*), 15 to 20% (4*S*)-KDGlu could be cleaved by *Ab*HpaI•Co^2+^ and *Ab*HpaI•Mn^2+^, while only 1% cleavage could be catalyzed by *Ab*HpaI•Zn^2+^. These results illustrate an interesting property of *Ab*HpaI•Zn^2+^ as a biocatalyst, because this form of enzyme exhibits strong stereoselectivity toward the 4*R*-isomer. Altogether, the data obtained from both (4*R*)-KDGal and (4*S*)-KDGlu cleavage reactions confirmed that *Ab*HpaI•M^2+^ prefers to cleave the 4*R*-isomer over 4*S* and the enzyme stereoselectivity is metal-dependent, with *Ab*HpaI•Zn^2+^ exhibiting the highest *R*/*S* stereoselectivity ratio, ∼5- to 9-fold higher than the Co^2+^-, Mn^2+^-, and Mg^2+^-bound *Ab*HpaI enzymes (Table 2).

#### Stereospecificity of AbHpaI aldol condensation

To explore whether *Ab*HpaI•M^2+^ enzymes have stereoselectivity toward the synthesis of the 4*R*-isomer in the aldol condensation reaction similar to the aldol cleavage reaction, we examined the product stereoisomer resulting from the condensation of pyruvate and D-glyceraldehyde catalyzed by *Ab*HpaI•M^2+^ enzymes. In order to differentiate between the two stereoisomeric compounds, (4*R*)-KDGal and (4*S*)-KDGlu, which have the same molecular mass of 178.0 g/mol, we used a high-sensitivity LC/MS system (Hi-Plex H cation-exchange column and triple quadrupole MS in a negative mode), which can distinguish between the two compounds (Fig. S3). Peak area of the product at *m/z* 177.0 ([M-H]^−^) was used for calculating the reaction yield.

The results obtained from aldol condensation of pyruvate (4 mM) and D-glyceraldehyde (30 mM) showed that only (4*R*)-KDGal was produced by all *Ab*HpaI•M^2+^ complexes of Zn^2+^, Co^2+^, Mn^2+^, and Mg^2+^, while the reaction of *Ab*HpaI•Ca^2+^ could not produce any product even after 60 min (Fig. 2*C* and Table 2). This was therefore confirmed that Ca^2+^ cannot promote pyruvate binding, consistent with the ITC binding result of pyruvate and *Ab*HpaI•Ca^2+^. Similar to the cleavage rates, the Co^2+^ containing enzyme catalyzes formation of (4*R*)-KDGal ≥ 2-fold faster than the Mn^2+^, Zn^2+^, and Mg^2+^-bound enzymes (Table 2). It should be noted that ∼0.1% (4*S*)-KDGlu formation could also be detected after 30 h (Fig. S4). The results clearly showed that *Ab*HpaI•M^2+^ prefers catalyzing stereoselective synthesis of the 4*R*-isomer, especially for the Zn^2+^-containing enzyme. Taken together, these findings show that *Ab*HpaI can practically catalyze stereospecific product formation.

#### Steady-state kinetics of AbHpaI•Zn^2+^

To understand the kinetics properties of the enzyme, steady-state kinetic parameters for aldol cleavage and condensation of *Ab*HpaI•Zn^2+^ were determined as listed in Table 3 and Fig. S5. Results indicate that the Michaelis–Menten constant (*K*_m_) of (4*R*)-KDGal cleavage was half of that for (4*S*)-KDGlu, suggesting that *Ab*HpaI•Zn^2+^ requires lower concentrations of (4*R*)-KDGal to reach the maximum velocity of the reaction. The *k*_cat_ value suggests that *Ab*HpaI•Zn^2+^ catalyzes the cleavage of (4*R*)-KDGal 18-fold faster than that of (4*S*)-KDGlu. In addition, the catalytic constant (*k*_cat_/*K*_m_) of (4*R*)-KDGal cleavage was about 36-fold greater than that of (4*S*)-KDGlu, indicating that *Ab*HpaI•Zn^2+^ catalyzes the aldol cleavage of (4*R*)-KDGal more efficiently than that of (4*S*)-KDGlu. These results agreed well with the activities measured in Table 2 in that *Ab*HpaI•Zn^2+^ is highly stereoselective toward 4*R*-isomer.

**Table 3:** Steady-state kinetics for aldol cleavage and condensation reactions of *Ab*HpaI•Zn^2+^

Substrate	Kinetic parameters		
	*k*_cat_ (min^−1^)	*K*_m_ (mM)	*k*_cat_/*K*_m_ (mM^−1^ min^−1^)
Aldol cleavage^a^			
(4*R*)-KDGal	3.0 ± 0.3	0.3 ± 0.1	10.0 ± 3.5
(4*S*)-KDGlu	0.17 ± 0.01	0.6 ± 0.1	0.3 ± 0.1
Aldol condensation of (4*R*)-KDGal synthesis^b^			
Pyruvate^c^	102.8 ± 15.9	1.6 ± 0.7	62.7 ± 29.2
D-Glyceraldehyde^d^	122.9 ± 17.6	10.6 ± 3.6	11.7 ± 4.3

a Kinetics of the cleavage reaction was investigated in buffer H containing 0.1 mM Zn^2+^, 0.05 to 2 mM (4*R*)-KDGal and 0.1 to 3.2 mM (4*S*)-KDGlu, and 5 or 40 μM *Ab*HpaI•Zn^2+^ (for (4*R*)-KDGal and (4*S*)-KDGlu, respectively). The amount of substrate was measured by RapidFire high-throughput mass spectrometry coupled with triple-quadrupole mass spectrometer.

b The condensation kinetics were analyzed by monitoring product formation using LC coupled with triple-quadrupole mass spectrometry.

c The reactions were carried out in 0 to 8 mM pyruvate with a fixed concentration of 30 mM D-glyceraldehyde. As pyruvate concentrations greater than 4 mM showed inhibition, the kinetics data at >4 mM pyruvate were not included in the analysis.

d Due to pyruvate inhibition at >4 mM, the reactions were carried out in 0 to 36 mM D-glyceraldehyde at 4 mM pyruvate.

For the aldol condensation kinetics, only the kinetics of (4*R*)-KDGal synthesis was investigated because (4*S*)-KDGlu could not be detected (Table 2). The results in Table 3 showed that the turnover number of (4*R*)-KDGal synthesis was ∼100 min^−1^ and the *K*_m_ value of pyruvate was approximately sevenfold lower than that of D-glyceraldehyde, indicative for higher affinity of pyruvate to the enzyme. Together, the kinetics results from both aldol cleavage and condensation reactions firmly support that *Ab*HpaI is stereoselective for the 4*R*-isomer. Further comparing the kinetics of (4*R*)-KDGal cleavage *versus* synthesis, it was interesting to note that the condensation reaction was much faster than the cleavage, as its turnover number was ∼40-fold greater. This property is interesting for *Ab*HpaI application as a biocatalyst because stereospecific aldol condensation is useful for preparation of APIs.

### Crystal structures of AbHpaI

#### The quaternary structure of AbHpaI is composed of a dimer of trimers

**Figure 3:**
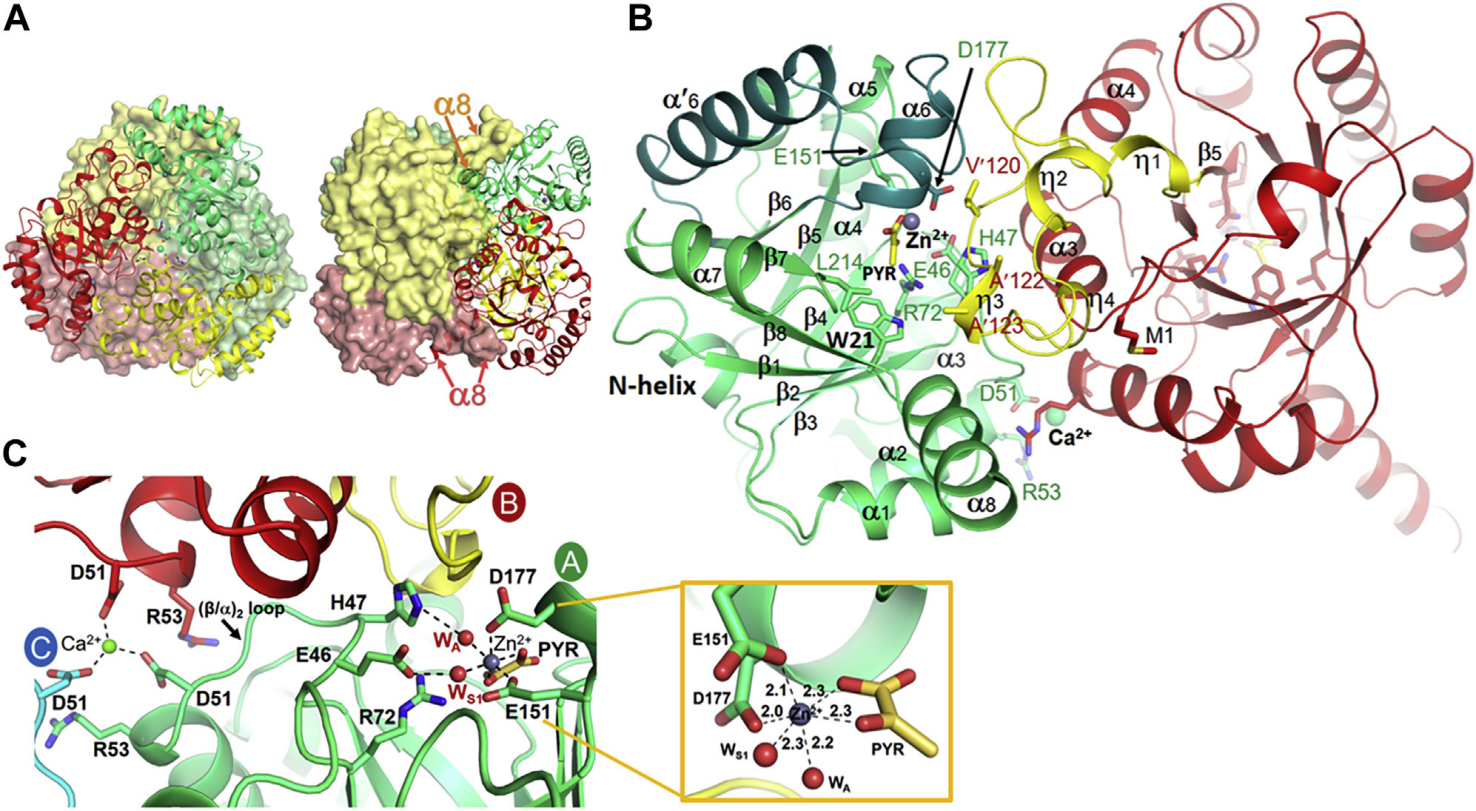
**X-ray structures of *Ab*HpaI.***A*, the quarternary hexameric structure of *Ab*HpaI is the result of dimerization of two trimers of *Ab*HpaI. One trimer is shown in ribbons docked on the second trimer shown as a surface model. *B*, the *Ab*HpaI dimer shows the subunit TIM barrel fold and the active pocket periphery—residues 110 to 136 in *yellow* contributed from the supporting subunit in *red*—of the active subunit in *green*. *C*, the pocket of Zn^2+^ and pyruvate (PYR) binding in Subunit A (*green*) is located at the interface between Subunit A and B (*red* and *yellow*). Two water molecules are drawn as *red spheres*. The *inset* shows the distances of Zn^2+^ with octahedral coordination in *Ab*HpaI•Zn^2+^•PYR. Ca^2+^ ion is bound at the *Ab*HpaI trimer neutralizing Asp51 negative charges. The distance between the catalytic Zn^2+^ and Ca^2+^ is 23.3 Å.

To gain insights into the molecular mechanism of *Ab*HpaI reactivity, we determined 11 crystal structures of *Ab*HpaI, including the apoenzyme, enzyme complexes Zn^2+^•(4*R*)-KDGal, Zn^2+^•(4*S*)-KDGlu and Mg^2+^•(4*R*)-KDGal (for understanding the aldol cleavage), and Co^2+^•pyruvate (PYR), Mn^2+^•PYR, Co^2+^•PYR•SSA, Mn^2+^•PYR•SSA, Zn^2+^•PYR•propionaldehyde (PPA), Zn^2+^•PYR•4-hydroxybenzaldehyde (HBA) (for understanding the aldol condensation) using molecular replacement method with *Ec*HpaI as a search template (PDB code 2V5J). *Ab*HpaI was crystallized in monoclinic *C*2 crystals, which diffracted at 1.85 to 2.0 Å resolutions. Data and refinement statistics are shown in Table 4 and electron density maps of ligands are shown in Figs. S6–S9. The crystal structure of *Ab*HpaI contains a trimer per asymmetric unit and a native hexameric quaternary structure can be drawn by applying twofold rotational symmetry (Fig. 3*A*). Size-exclusion chromatography (SEC) also confirmed a hexameric form of *Ab*HpaI (Fig. S10).

**Table 4:** Data collection and refinement statistics of *Ab*HpaI complexes

Parameters	Apo	Zn^2+^•Pyr	Co^2+^•Pyr^a^	Mn^2+^•Pyr^a^	Zn^2+^•(4*R*)-KDGal	Zn^2+^•(4*S*)-KDGal	Mg^2+^•(4*R*)-KDGal	Mn^2+^•Pyr•SSA	Co^2+^•Pyr•SSA	Zn^2+^•Pyr•PPA	Zn^2+^•Pyr•HBA
PDB code	7ET8	7ET9	7ETA	7ETB	7ETC	7ETD	7ETE	7ETF	7ETG	7ETH	7ETI
Data Collection											
Resolution (Å)	20.79–1.90 (2.00–1.90)	30.00–1.90 (1.97–1.90)	30.00–1.85 (1.92–1.85)	30.00–1.85 (1.92–1.85)	24.38–1.95 (2.05–1.95)	24.43–1.90 (2.00–1.90)	24.40–1.90 (2.00–1.90)	20.65–2.00 (2.10–2.00)	21.12–1.90 (2.00–1.90)	20.85–2.20 (2.30–2.20)	24.42–1.95 (2.05–1.95)
Wavelength (Å)	1.54	1.54	1.54	1.54	1.54	1.54	1.54	1.54	1.54	1.54	1.54
Space group	*C*2	*C*2	*C*2	*C*2	*C*2	*C*2	*C*2	*C*2	*C*2	*C*2	*C*2
Unit cell (Å)											
*a*, *b*, *c* (Å)	*a* = 147.620, *b* = 90.163, *c* = 86.484	*a* = 147.811, *b* = 89.646, *c* = 86.464	*a* = 147.359, *b* = 90.323, *c* = 86.440	*a* = 147.724, *b* = 90.345, *c* = 86.600	*a* = 147.36, *b* = 90.31, *c* = 86.39	*a* = 147.22, *b* = 90.52, *c* = 86.41	*a* = 147.56, *b* = 90.35, *c* = 86.52	*a* = 147.25, *b* = 89.29, *c* = 86.14	*a* = 147.75, *b* = 89.69, *c* = 86.30	*a* = 147.18, *b* = 89.64, *c* = 86.31	*a* = 147.37, *b* = 90.49, *c* = 86.50
β (°)	122.325	122.735	122.093	122.26	122.2	122.0	122.2	122.7	122.5	122.6	122.1
Total Reflections	402,575	585,449	665,519	679,987	417,647	702,725	598,751	470,541	677,459	426,770	666,758
Unique Reflections	74,776	73,606	81,180	81,847	68,875	75,801	75,392	60,695	74,655	47,950	69,969
Completeness (%)	99.1 (97.6)	98.3 (88.1)	99.0 (91.0)	99.6 (96.8)	98.6 (98.4)	99.1 (99.9)	99.6 (100)	95.7 (83.5)	99.8 (99.4)	99.7 (100)	99.8 (100)
Average *<I/σ>*	13.85 (4.56)	25.51 (3.10)	21.15 (1.85)	22.17 (3.17)	11.5 (3.3)	14.5 (4.4)	13.4 (3.7)	19.0 (5.1)	15.1 (3.3)	13.8 (5.0)	15.5 (4.2)
*R*_*meas*_	0.098 (0.369)	0.083 (0.467)	0.075 (0.343)	0.076 (0.149)	0.137 (0.697)	0.147 (0.588)	0.131 (0.719)	0.072 (0.260)	0.093 (0.431)	0.160 (0.529)	0.117 (0.698)
*CC*_*1/2*_	0.995 (0.845)	0.998 (0.847)	0.974 (0.856)	0.987 (0.909)	0.992 (0.819)	0.994 (0.879)	0.995 (0.861)	0.998 (0.966)	0.998 (0.970)	0.992 (0.874)	0.997 (0.890)
Refinement											
*R*_*f*_/*R*_*free*_ (%)	17.36/20.16	15.11/17.03	16.17/18.64	15.09/17.64	18.4/21.8	16.4/18.7	17.5/19.8	18.8/22.3	19.1/21.9	18.0/21.7	17.0/20.3
Protomers/ASU	3	3	3	3	3	3	3	3	3	3	3
No. atoms/*B*-factor (Å^2^)											
Protein	5805/15.4	5805/13.7	5781/15.2	5781/17.2	5781/15.3	5781/13.8	5781/15.9	5781/15.7	5781/18.4	5736/13.0	5781/14.6
Zn^2+^/Mn^2+^/Co^2+^/Mg^2+^	-	3/12.3	3/11.2	3/13.0	3/17.9	3/16.6	3/35.4	3/18.4	3/15.0	3/13.9	3/11.7
Pyruvate (Pyr)	-	18/14.7	18/10.9	18/12.8	12/22.2	-	-	18/15.8	18/14.6	18/20.7	18/13.1
(4*R*)-KDGal	-	-	-	-	12/30.6	-	36/42.7	-	-	-	-
(4*S*)-KDGlu	-	-	-	-	-	36/33.4	-	-	-	-	-
SSA/PPA/HBA	-	-	-	-	-	-	-	21/46.0	21/42.8	12/26.6	27/36.2
Ca^2+^	1/15	1/23.9	1/18.5	1/9.9	1/17.9	1/15.6	1/14.9	1/41.4	1/54.1	1/42.5	1/17.5
Water	639/24.3	484/19.9	499/20.5	701/24.8	374/18.1	721/23.4	655/24.6	385/20.3	502/26.1	280/15.3	586/22.0
Rms deviation											
Bond length (Å)	0.011	0.009	0.010	0.012	0.008	0.007	0.008	0.011	0.011	0.009	0.009
Bond angle (°)	1.672	1.571	1.461	1.685	1.476	1.415	1.491	1.569	1.599	1.536	1.470
Ramachandran Plot											
Favored (%)	93.5	93.5	94.3	94.0	91.7	93.4	93.8	92.5	94.3	93.2	92.6
Allowed (%)	6.5	6.5	5.7	6.0	8.3	6.6	6.2	7.5	5.7	6.8	7.4
Outlier (%)	0	0	0	0	0	0	0	0	0	0	0

Values in parentheses are for the highest resolution shells. *R*_f_ = Σ_hkl_||*F*_obs_| − |*F*_calc_||/Σ_hkl_|*F*_obs_|, where *F*_obs_ and *F*_calc_ are the observed and calculated structure-factor amplitudes, respectively. *R*_free_ was calculated in the same manner as *R*_f_ but using only a 10% unrefined subset of the reflection data.

a Data were processed with Proteum3 except datasets of Co^2+^•Pyr and Mn^2+^•Pyr using HKL2000.

Each protomer contains eight β/α motifs of a TIM barrel fold with an additional α-helix (residues 6–11; N-helix) at the N-terminus (Fig. 3*B*). Three protomers are associated in a tight trimeric structure to generate three catalytic pockets, and the trimer is dimerized to form a stable and rigid hexamer with 35% buried area. The hexameric quaternary structure of *Ab*HpaI is stabilized through extensive interactions from α-helices of the (β/α)_1_ (residues 27–38), (β/α)_2_ (residues 53–64), and (β/α)_8_ (residues 236–253). The α-helix (residues 236–253) of the (β/α)_8_ of each protomer docks on the surface of the neighboring trimer for holding the trimer of dimers. In the structures of *Ab*HpaI, a C-terminal end at residues 254 to 266 was not built because of no electron density. Each active site, located on a side face of the trimer, was built from two protomer subunits with a main catalytic pocket located in one subunit accompanied by a loop linker of residues 110 to 136 between (β/α)_4_ and (β/α)_5_ motifs of the shared protomer as a pocket periphery (Fig. 3*B*).

#### Metal ion octahedral coordination in AbHpaI is important for AbHpaI reactivity and stability

Understanding how *Ab*HpaI accommodates the M^2+^ cofactor could yield biochemical insights into substrate recognition, reactivity, and stereospecificity control by different types of metal ions. Therefore, the coordination geometry of the M^2+^ cofactor in each complex obtained was analyzed. The crystal structures of *Ab*HpaI complexed with metal ions Zn^2+^, Co^2+^, or Mn^2+^ and pyruvate (PDB codes 7ET9, 7ETA and 7ETB) revealed all types of M^2+^ chelate to a carboxyl group of Asp177, a water molecule (W_S1_), and a carboxyl and 2-oxo groups of pyruvate in a square planar arrangement, and with a carboxyl group of Glu151 and a water molecule (W_A_) in an axial position, arranged in an octahedral coordination geometry (Fig. 3*C*). Besides Glu151 and Asp177, Glu46 and His47 on the (β/α)_2_ loop also provide water-mediated hydrogen bondings *via* W_A_ and W_S1_. Superposition of all *Ab*HpaI complex structures revealed that the bound M^2+^ cofactor is at the same position with six atoms in octahedral geometry.

Notably, Glu151 and Asp177 in the Zn^2+^, Co^2+^, or Mn^2+^ complexes were more rigid than those of apo-*Ab*HpaI, as reflected by temperature factors (B-factors) of the crystal structure. In the *Ab*HpaI•M^2+^ complex structure, the B-factors of the (β/α)_4_ and (β/α)_6_ loop regions including the helix α_6_ where Asp177 is located are smaller than that of the apo structure. This suggests that M^2+^ can reduce the mobility in this region and strengthen subunit compactness, thereby stabilizing the overall structural architecture. In addition, the formation of the M^2+^ octahedral coordination with pyruvate in *Ab*HpaI is important for enzyme reactivity.

#### Ca^2+^ ion neutralizing negatively charged Asp51 at the AbHpaI trimer surface facilitates dimerization of subunits

Ca^2+^ ion was found abundantly in purified *Ab*HpaI (Fig. 1*A*). However, it does not act as a cofactor to enhance enzyme catalysis (Table 2). Therefore, the function of Ca^2+^ ion in *Ab*HpaI was further investigated by analyzing the enzyme structure in complex with Ca^2+^.

The crystal structure of apo-*Ab*HpaI (PDB code 7ET8) crystallized in the presence of CaCl_2_ only showed Ca^2+^ at the defined trimer center (not in the active site) in a distorted octahedral geometry with three Asp51 side chains (2.3–2.4 Å) and three water molecules as observed in all structures of *Ab*HpaI studied here (Figs. 3*C* and S9). These data, together with the nonfunctional role of Ca^2+^ discussed above, confirmed that Ca^2+^ does not serve as a cofactor, but rather acts as a stabilizing factor on the dimerization surface of the *Ab*HpaI trimer by neutralizing the negative charges of Asp51 on the trimer surface. In *Ec*HpaI, the equivalent position to Asp51 was found to be Asn (Asn48^*Ec*^), thus abolishing the ability of this enzyme to bind to a divalent metal ion. Therefore, Ca^2+^ functions to prevent repulsive forces and to reduce movement of the (β/α)_2_ loop in *Ab*HpaI where Asp51 sits. Consequently, by stabilizing the (β/α)_2_ loop where the active residues Glu46 and His47 reside (see proposed mechanisms), Ca^2+^ could indirectly aid in the catalysis of *Ab*HpaI.

#### Insights into stereoselectivity of AbHpaI in the aldol cleavage reaction

**Figure 4:**
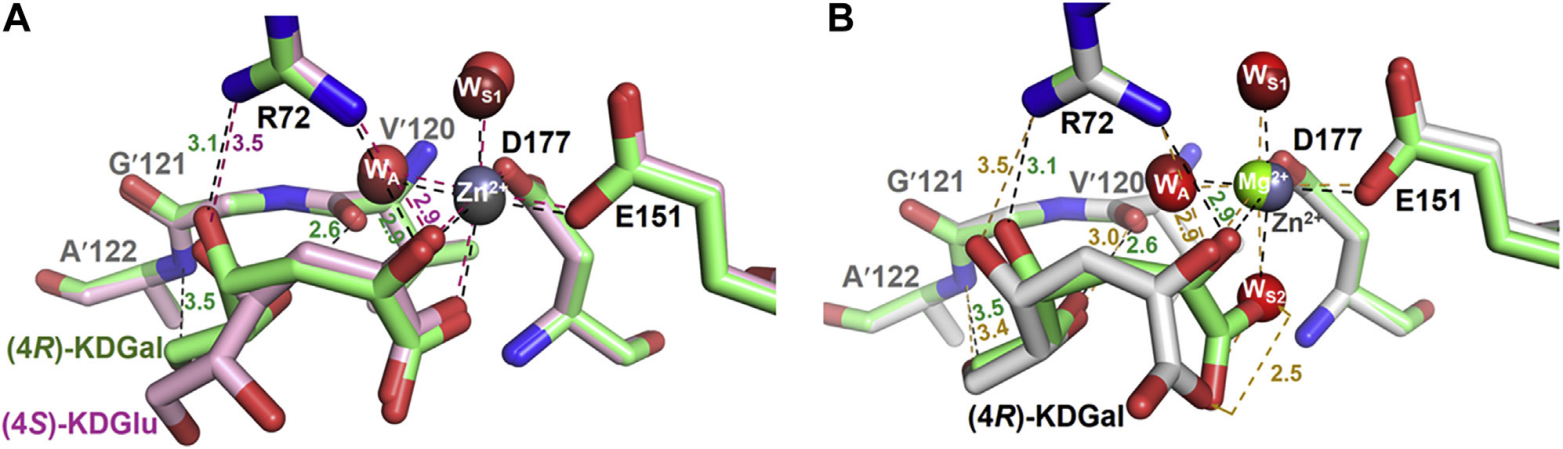
**Binding interactions of ligands in the active site of *Ab*HpaI.** Binding features of (4*R*)-KDGal and (4*S*)-KDGlu show differences in the stabilization interactions among the complexes. Superposition of *Ab*HpaI•Zn^2+^•(4*R*)-KDGal (PDB code 7ETC) in *green* with (*A*) *Ab*HpaI•Zn^2+^•(4*S*)-KDGlu (PDB code 7ETD) in *pink*, and (*B*) *Ab*HpaI•Mg^2+^•(4*R*)-KDGal (PDB code 7ETE) in *white*. In (*A*), the C_4_-OH of (4*R*)-KDGal is hydrogen bonded with Arg72 (3.1 Å), whereas the C_4_-OH of (4*S*)-KDGlu is in longer distance (3.5−3.6 Å), making it less susceptible for C_3_–C_4_ bond cleavage. There are also differences in the interactions from the supporting subunit in which the C_5_-OH and C_6_-OH of (4*R*)-KDGal are directly stabilized by hydrogen bonding with Val′120 and Ala′122, while the hydroxyl groups of (4*S*)-KDGlu are more than 6 Å away from these residues. In (*B*), binding interactions of Mg^2+^•(4*R*)-KDGal are quite similar to the Zn^2+^•(4*R*)-KDGal except that the COOH in the Mg^2+^•(4*R*)-KDGal complex does not directly chelate Mg^2+^, but it is replaced by water (W_S2_), which is mediated by hydrogen bonding *via* the Mg^2+^ octahedral coordination.

To gain insights into why *Ab*HpaI significantly prefers the 4*R*-isomer over the 4*S*-isomer in the aldol cleavage reaction (Table 2), crystal structures of *Ab*HpaI•Zn^2+^ in complex with (4*R*)-KDGal (PDB code 7ETC) and (4*S*)-KDGlu (PDB code 7ETD) were determined at 1.95 and 1.90 Å resolutions, respectively. Superimposed structures illustrated that for the pyruvate core, the 1-carboxyl and 2-oxo groups of both substrates are directly coordinated to the Zn^2+^ site in an octahedral geometry similar to that found during pyruvate binding (Figs. 3*C* and 4*A*). However, the major differences are at the C_4_-OH, which interacts with Arg72 and at the binding site of the D-glyceraldehyde moiety. The structures revealed that the 4-OH of (4*R*)-KDGal forms a hydrogen bond with a guanidinium side chain of Arg72 at a 3.1 Å distance, whereas that of (4*S*)-KDGlu interacts with a longer distance (3.5–3.6 Å) (Fig. 4*A*). This therefore affected the arrangement and interactions of the D-glyceraldehyde moieties in the two compounds. The 5-OH and 6-OH functional groups of the D-glyceraldehyde moiety of (4*R*)-KDGal form hydrogen bonds with the main chains of Val′120 (2.6 Å) and Ala′122 (3.5 Å) from the pocket site created by the neighboring subunit, while those of (4*S*)-KDGlu do not form such interactions. Clearly, the observed interaction differences implied that *Ab*HpaI•Zn^2+^ could preferably bind (4*R*)-KDGal over (4*S*)-KDGlu, further supported by QM/MM MD simulations. The binding energies of (4*R*)-KDGal and (4*S*)-KDGlu were calculated as −158 ± 9 and −131 ± 8 kcal/mol, respectively (Table S2). Furthermore, the configuration of (4*R*)-KDGal bound in the *Ab*HpaI•Zn^2+^ complex can provide more suitable orientation and decreased motion of the substrate for the aldol cleavage in contrast to the bound (4*S*)-KDGlu. The structural analysis clearly supports the cleavage activity of the Zn^2+^-bound enzyme toward the 4*R* over the 4*S* substrates (Tables 2 and 3).

In addition, to understanding why the Mg^2+^ cofactor gave such a slow cleavage rate, the crystal structure of the *Ab*HpaI•Mg^2+^•(4*R*)-KDGal complex (PDB code 7ETE) solved at 1.95 Å resolution was compared with the structure of the *Ab*HpaI•Zn^2+^•(4*R*)-KDGal complex. Superimposed structures revealed a significant difference at the pyruvate core linked to the metal ion cofactors (Fig. 4*B*). A water W_S2_ replaced the carboxyl group of (4*R*)-KDGal to join the Mg^2+^ octahedral coordination. This feature gives rise to a longer distance between the 4-OH and Arg72 side chain in the Mg^2+^ complex (3.4−3.6 Å), causing a weaker binding interaction compared with the Zn^2+^ complex, thereby decelerating the C_3_–C_4_ bond breakage (Fig. 4*B* and Table 2). Based on structural and kinetics analyses, it could be summarized that the key binding features of the ligand important for stereoselective control in the aldol cleavage reaction of *Ab*HpaI were (i) C_4_-OH anchoring by Arg72, (ii) interaction of the substrate pyruvate moiety in an octahedral M^2+^ complex, and (iii) interactions of the aldehyde moiety with the neighboring subunit.

### Biocatalytic aspects of AbHpaI

#### AbHpaI catalyzes the aldol condensation reactions with broad aldehyde specificity

To investigate whether *Ab*HpaI•Zn^2+^ can use various aldehydes as substrates in aldol condensation with pyruvate, we screened different categories of aliphatic and aromatic aldehydes. The corresponding aldol products were analyzed using liquid chromatography with high-resolution mass spectrometry to measure the exact *m/z* in a negative mode. The results showed that the selected aldehydes could be used by *Ab*HpaI•Zn^2+^ in aldol condensation with pyruvate to yield various products (*m/z* values shown in Tables 5 and S3, and Fig. S11). The derivatives of aliphatic aldehydes with C_3_–C_6_ chain length could be successfully converted into the corresponding 4-hydroxy-2-keto aliphatic acids (Tables 5 and S3, and Fig. S11). Next, we examined with aromatic aldehydes and found that *Ab*HpaI•Zn^2+^ can catalyze aldol condensation of pyruvate with various aromatic aldehydes such as benzaldehyde, HBA, and anisaldehyde, to yield the corresponding 4-hydroxy-2-keto aromatic acids (Tables 5 and S3, and Fig. S11). From our data, *Ab*HpaI•Zn^2+^ can use a wide range of aldehyde substrates, suggesting that the enzyme active site is flexible enough to accommodate a variety of aldehydes for aldol condensation.

#### Space for accommodating various aldehydes in the active site of AbHpaI

To understand how the *Ab*HpaI can accommodate various aldehydes for aldol condensation, we investigated the binding interactions of three aldehydes from the cocrystal structures of *Ab*HpaI•Mn^2+^•PYR and *Ab*HpaI•Co^2+^•PYR with SSA (PDB codes 7ETF and 7ETG, respectively), and *Ab*HpaI•Zn^2+^•PYR with PPA and HBA (PDB codes 7ETH and 7ETI, respectively).

**Figure 5:**
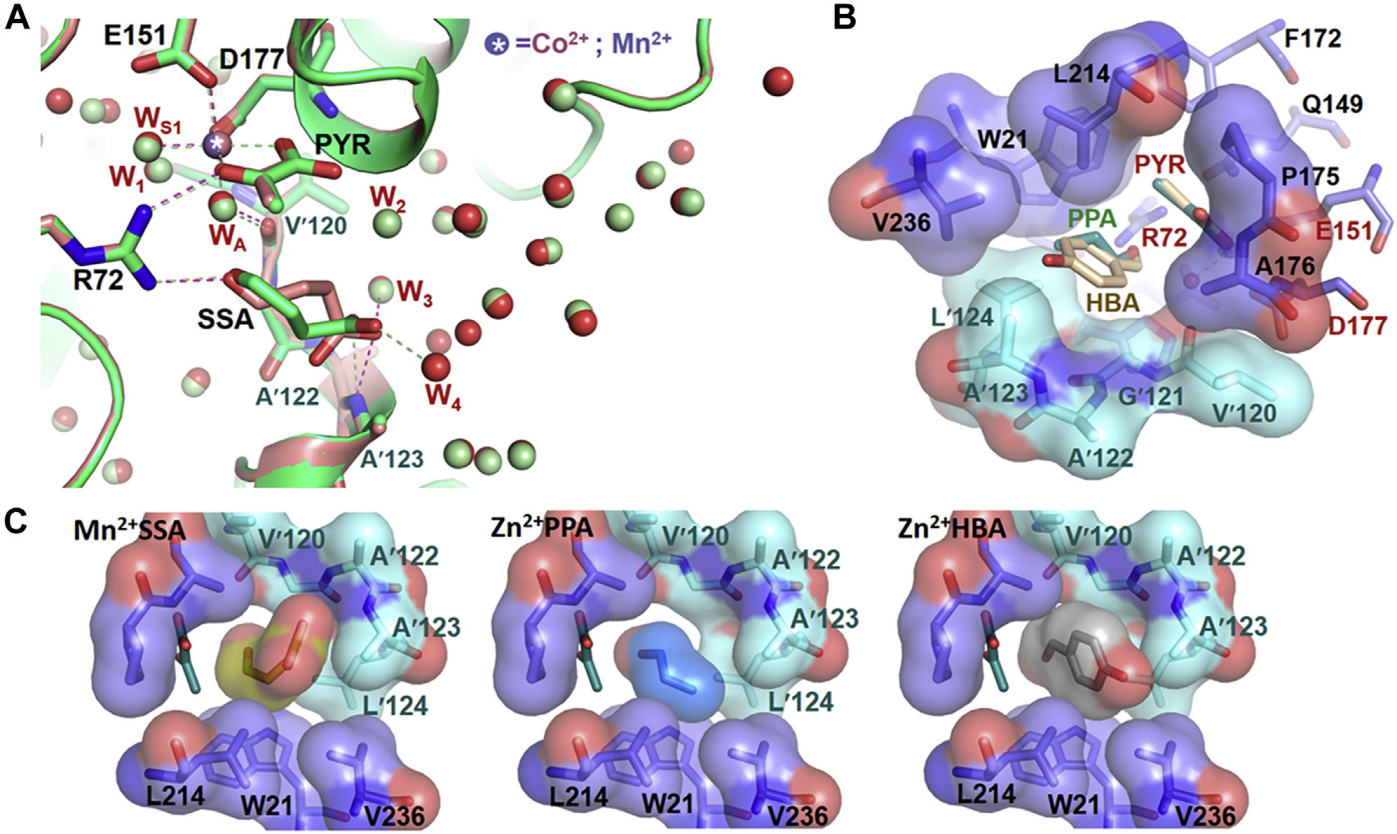
**Space for accommodating aldehyde binding in *Ab*HapI.** The X-ray structures of *Ab*HpaI complexes of (*A*) Mn^2+^•PYR•SSA (*green*) and Co^2+^•PYR•SSA (*pink*) show the active binding site located at the *bottom* of the binding pocket and hydrogen bonding interactions between *Ab*HpaI with PYR and SSA (*dashed lines*) in a wide pocket filled with water molecules shown as *spheres* (*green* for the Mn^2+^ and *red* for Co^2+^ complexes). Water molecules filled in the binding pocket provide water networks for substrate stabilization. *B*, overlaid structures of Zn^2+^•PYR•PPA and Zn^2+^•PYR•HBA show binding mode of PPA and HBA. *C*, surface representatives of *Ab*HpaI•M^2+^•PYR•aldehyde structures show van der Waals spheres between pocket residues and the aldehyde substrates SSA, PPA, and HBA with PYR in *green stick* representation.

**Figure 6:**
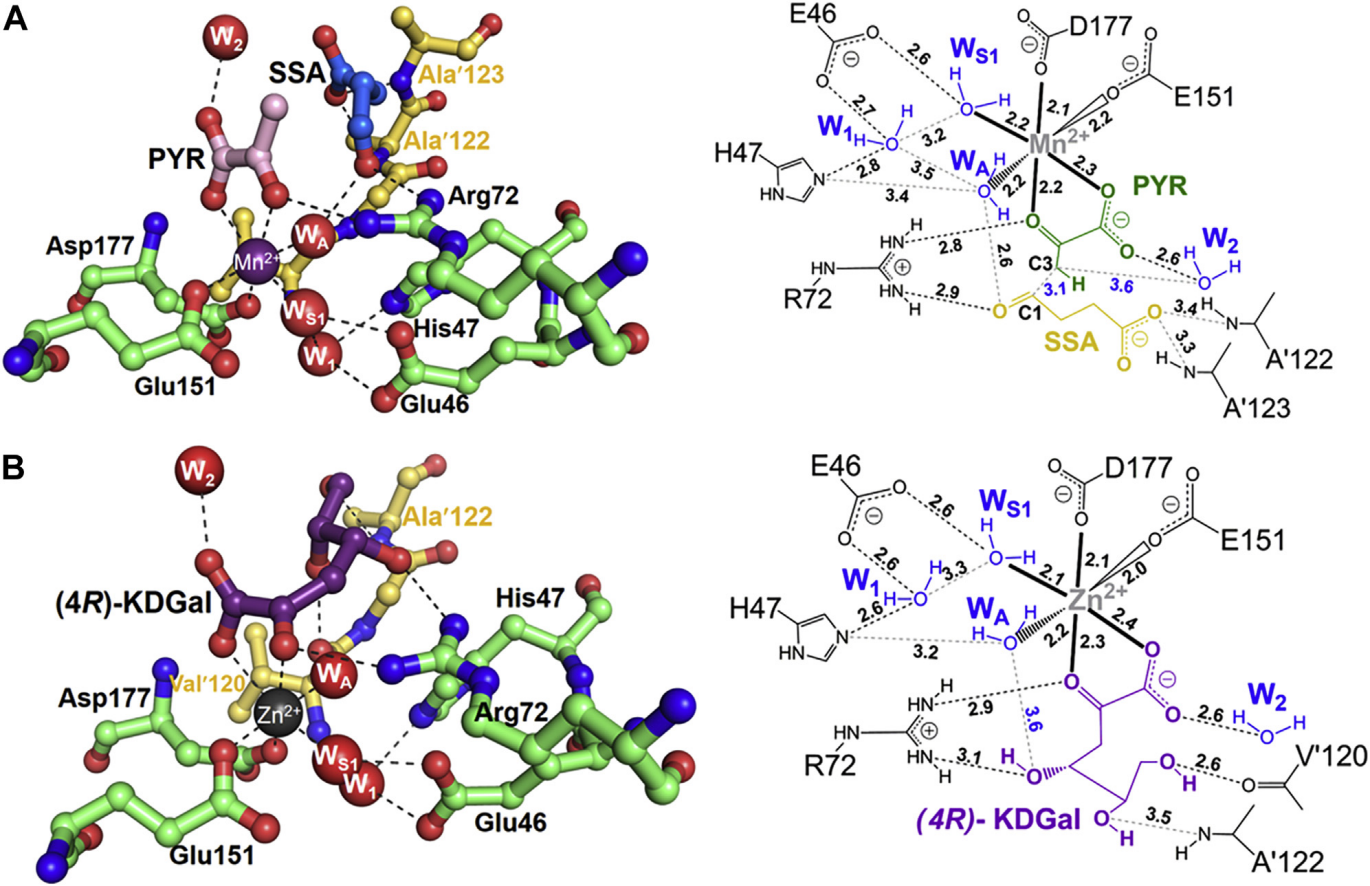
**Key residues and structural waters at the active site.***A*, *Ab*HpaI•Mn^2+^•PYR•SSA (PDB code 7ETF) and (*B*) *Ab*HpaI•Zn^2+^•(4*R*)-KDGal (PDB code 7ETC) structures are presented on the *left* and a simple schematic diagram of each structure is on the *right*. Water molecules are labeled as W_A_, W_S1_, W_1_, and W_2_. *Dash lines* display H-bonds and distances between atoms. H-bond distances are designated in *black digits*, while the distances between W_A_ to 4-OH of (4*R*)-KDGal and W_2_ to C_3_ of PYR and C_1_ of SSA to C_3_ of PYR are in *blue*.

Overlaid structures of *Ab*HpaI•Mn^2+^•PYR•SSA and *Ab*HpaI•Co^2+^•PYR•SSA revealed that at the aldehyde binding site, the carbonyl group of SSA forms hydrogen bonds with Arg72 (2.7−2.9 Å) and the apex W_A_ water (2.6–2.9 Å) in the M^2+^ octahedral coordination, while the carboxyl tail of SSA is hydrogen bonded to the Ala′123 or Ala′122 NH backbone of the nearby subunit and to a water network *via* W_3_ or W_4_ in the pocket tunnel filled with waters (Figs. 5*A* and 6*A*, see later). In addition, a distance between the C_1_ atom of SSA and the C_3_ methyl group of PYR was in the range of 3.1 to 4.1 Å (Fig. 6*A*, see later). We found that both metal ion complexes provide a similar binding of PYR but a slightly different configuration of SSA (Fig. 5*A*). This indicated that space for accommodating aldehyde substrate is larger than a van der Waals sphere of SSA, hence with a flexible hydrocarbon backbone, two configurations of SSA can be docked (Fig. 5*A*).

Although the substrate binding pocket of *Ab*HpaI is wide open and exposed to outside solvent on the protein surface, the opening narrows down to the bottom of the active site where the reaction takes place. The site for aldehyde docking appeared to be hydrophobic, as most of the residues lining the site are nonpolar except for Arg72 (Fig. 5). Next, we explored the crystal structure of the *Ab*HpaI•Zn^2+^•PYR complex liganded with PPA, which is a more hydrophobic ligand than SSA. PPA, which has only a polar carbonyl moiety, can dock on a hydrophobic cleft, composed of Trp21, Leu214, Val236 of the active subunit, and Leu′124 from the shared subunit (Fig. 5, *B* and *C*) and arranges the carbonyl moiety to hydrogen bond with Arg72 in a reactive trajectory for condensation with PYR, while the pocket tunnel is still full of water. However, the number of long-chain hydrocarbons in the aldehydes may be limited due to substrate solubility if the reaction is carried out solely in an aqueous environment because the long-chain hydrocarbon would protrude out of the hydrophobic cleft toward the water milieu in the direction similar to the SSA trajectory (Fig. 5, *A* and *C*). Thus, the nonpolar aldehydes may not be able to move along the water tunnel to reach the catalytic site located deep within the protein. For aromatic aldehydes, HBA was chosen as a representative to explore the binding mode. The cocrystal structure of the *Ab*HpaI•Zn^2+^•PYR•HBA complex shows that the Trp21 indole ring, Leu214, and Val236 stabilize the HBA benzene ring through van der Waals interactions, while the carbonyl moiety forms a hydrogen bond to Arg72, which may be crucial for aldol condensation with PYR (Fig. 5, *B* and *C*). This structural analysis confirmed that *Ab*HpaI can accommodate aromatic aldehydes well, as long as they can pass through a polar environment to get inside the active pocket.

#### Proposed mechanism for aldol reactions catalyzed by AbHpaI

**Figure 7:**
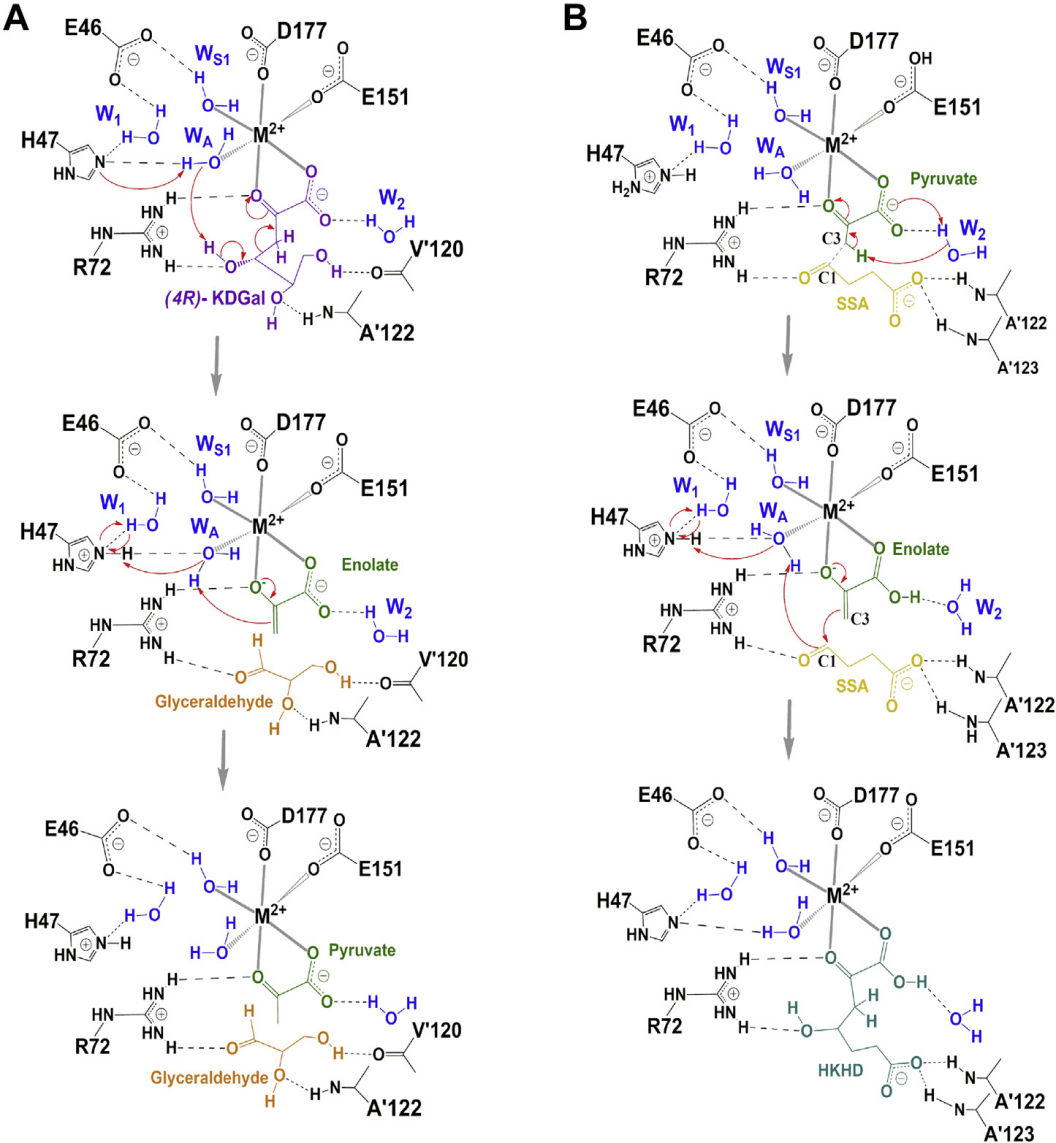
**Proposed mechanisms for aldol reactions catalyzed by *Ab*HpaI.** The minimal schemes for (*A*) aldol cleavage of (4*R*)-KDGal were proposed based on *Ab*HpaI•Zn^2+^•(4*R*)-KDGal (PDB code 7ETC) and for (*B*) aldol condensation of pyruvate and SSA to yield HKHD was proposed based on *Ab*HpaI•Mn^2+^•PYR•SSA (PDB code 7ETF). The key residues Glu46, His47, and Arg72 together with bound water molecules W_A_, W_S1_, W_1_, and W_2_ facilitate aldol cleavage and condensation reactions. The aldol cleavage mechanism begins with a cascade of proton abstraction mediated by His47 and W_A_, general acid/base. The abstraction of a C_4_-OH proton mediated by W_A_ results in a C_3_–C_4_ bond cleavage of (4*R*)-KDGal, which can be stabilized by Arg72, to generate D-glyceraldehyde and enolate. The enolate intermediate then abstracts a proton from W_A_, which can be facilitated by His47 and nearby H-bond networks of W_1_, Glu46, and W_S1_. For the aldol condensation mechanism, W_2_ water mediated by the pyruvate carboxylate anion is proposed to act as a catalytic base to abstract a proton from the C_3_-methyl group of pyruvate to yield an enolate intermediate. The carbanion C_3_ of the enolate attacks the C_1_ of SSA to yield HKHD, followed by a protonation step from W_A_ to yield a C_4_-OH.

The results from structural analysis reveal several structural water molecules at the active site potentially involved in *Ab*HpaI catalysis (Fig. 6). A possible model for the *Ab*HpaI aldol cleavage mechanism is proposed in Figure 7*A*. The cleavage of a C_3_–C_4_ bond in the (4*R*)-KDGal substrate to yield pyruvate and D-glyceraldehyde products is facilitated by Glu46, His47, and Arg72 together with bound water molecules, W_A_, W_1_, and W_S1_, to deprotonate the 4-OH leading to the bond cleavage to form an enolate intermediate, which then abstracts a proton from W_A_ to yield a pyruvate.

For aldol condensation, *via* pyruvate carboxylate mediation, a water W_2_ likely acts as a catalytic base to abstract a proton from the C_3_-methyl of pyruvate in a similar reaction to that of Glu46, His47, and Arg72 with three waters W_A_, W_1_, and W_S1_ to generate the enolate intermediate, which then forms a covalent linkage with the C_1_ atom of SSA to produce HKHD (Fig. 7*B*). This model is supported by a p*K*_a_ value of pyruvate C_3_-methyl of ∼6.5 (previously estimated by a pD-profile of pyruvate C_3_ proton exchange reaction of *Ec*HpaI (39)).

#### Thermal and solvent stability

**Figure 8:**
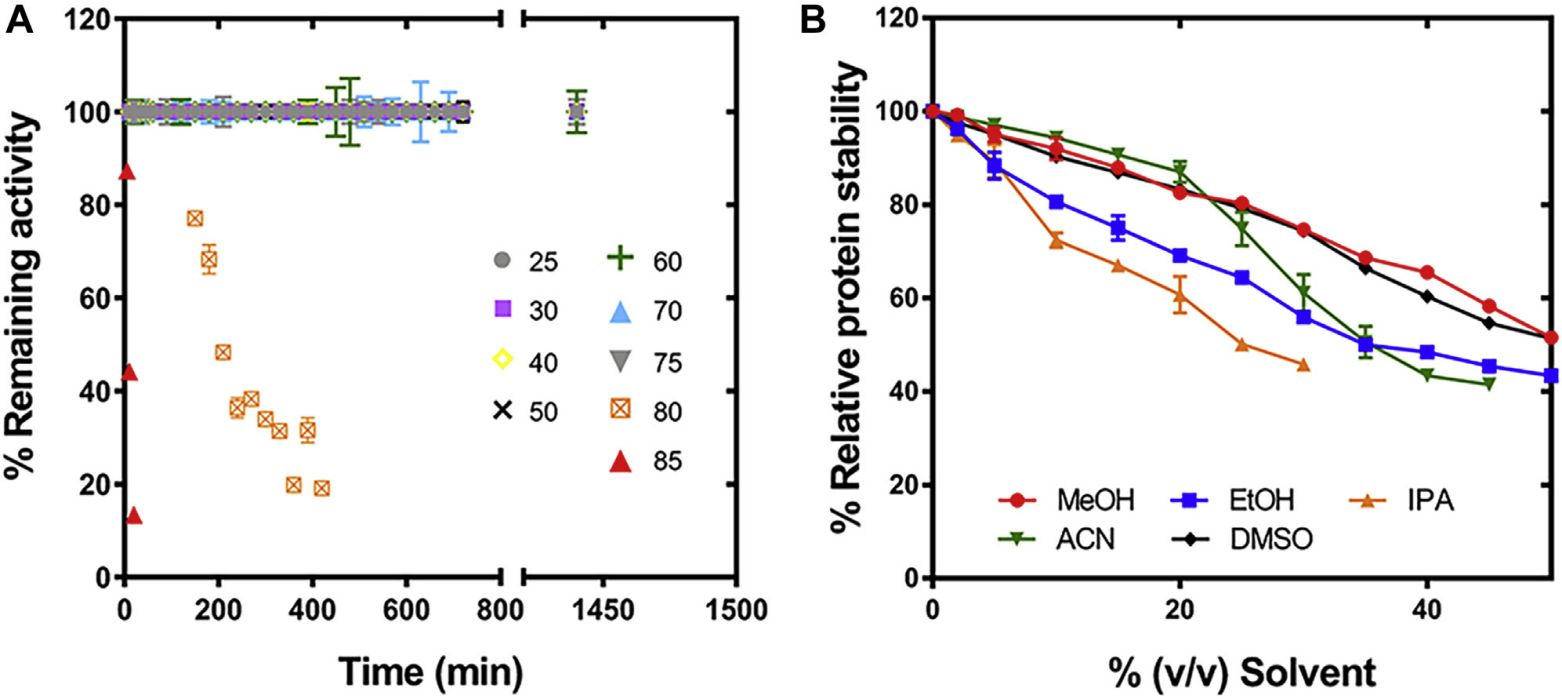
**Thermostable and solvent-tolerant properties of *Ab*HpaI.***A*, thermostability of *Ab*HpaI•Zn^2+^ was investigated by measuring *Ab*HpaI•Zn^2+^ activities remained by LDH-coupled assays upon incubation of the enzyme at various temperatures ranging from 25 to 85 °C for various periods. *B*, plots of *Ab*HpaI•Zn^2+^ protein stability in the presence of different % (v/v) organic solvents (MeOH, EtOH, IPA, can, and DMSO). Percentage of relative protein stability was calculated from the *T*_*m*_ of *Ab*HpaI in the absence of solvents as 100%. *Error bars* represent standard deviations (S.D.) from three replications of the data.

Thermostability and organic solvent tolerance are important requirements for biocatalytic applications (42). Therefore, we determined the effect of M^2+^ on the thermostability of *Ab*HpaI using thermofluor stability measurements. The results showed that apo-*Ab*HpaI is quite thermostable naturally with a protein melting temperature (*T*_m_) value as high as 81.3 °C. The binding of transition M^2+^, but not alkaline earth M^2+^, can further increase the thermostability of apo-*Ab*HpaI (Table 1). The *T*_m_ values of *Ab*HpaI were enhanced by 5.7, 3.4, and 1.7 °C upon binding to Zn^2+^, Co^2+^, and Mn^2+^, respectively. In contrast, the change of *T*_m_ values for *Ab*HpaI•Mg^2+^ and *Ab*HpaI•Ca^2+^ was negligible. Consequently, the result suggested that Zn^2+^ enhances the thermostability of *Ab*HpaI, the highest among all types of *Ab*HpaI•M^2+^ enzymes. Apart from the *T*_m_ value, which represents the protein stability, we also measured the remaining activity of *Ab*HpaI•Zn^2+^ incubated at increasing temperatures (25–85 °C) for various incubation times (0–24 h) to examine the thermostability of *Ab*HpaI. The result in Figure 8*A* showed that *Ab*HpaI•Zn^2+^ can tolerate a wide range of temperatures from 25 to 75 °C for 24 h (and possibly longer) and can tolerate 80 °C for up to 2 h without activity loss.

We then explored the effects of Zn^2+^ on solvent tolerance of *Ab*HpaI. As most substrates of aldolase reactions such as aliphatic and aromatic aldehydes are not soluble well in aqueous phases, addition of organic solvents is required to enhance substrate solubility. Therefore, we determined the *T*_*m*_ of *Ab*HpaI•Zn^2+^ complex in the presence of organic solvents to represent enzyme stability. The organic solvents generally used in industries were chosen in this study, namely polar-protic (methanol, MeOH; ethanol, EtOH; and isopropanol, IPA) and polar-aprotic (acetronitrile, ACN; and dimethylsulfoxide, DMSO). The *T*_*m*_ results showed that upon addition of 20% (v/v) MeOH, ACN, and DMSO, the protein stability of *Ab*HpaI was perturbed by only 10% (Fig. 8*B*), while EtOH and IPA disrupted protein stability by about 30 to 40%. This implied that 20% (v/v) of MeOH, can, or DMSO can be used as a cosolvent to enhance substrate solubility with small perturbation in protein stability of *Ab*HpaI. Taken together, these findings suggest that *Ab*HpaI is thermostable and solvent-tolerant enzyme, which can be a promising robust biocatalyst for aldol reaction.

## Discussion

Our report here has shown that *Ab*HpaI is distinct among pyruvate-specific Class II metal aldolases for its ability to catalyze stereospecific aldol condensation and to use Zn^2+^ as a cofactor. Zn^2+^ binding can enhance stereoselectivity in aldol reactions and enzyme thermostability. Comprehensive structural investigation of *Ab*HpaI complexes can explain how the enzyme is more stereoselective toward (4*R*)-KDGal over (4*S*)-KDGlu and how a variety of aldehydes can be accommodated.

Zn^2+^ is the most abundant transition metal ion found in the purified *Ab*HpaI, binds with the highest affinity, and significantly increases the substrate stereoselectivity and stability of *Ab*HpaI (Figs. 1, 2, 4, 8, S4, S5 and Table 1, Table 2, Table 3, S2). In addition, the enzyme can use other divalent ions (Co^2+^, Mn^2+^, and Mg^2+^) as cofactors, but these metal ions do not mediate *Ab*HpaI stereoselective aldol cleavage functions as effectively as Zn^2+^. This property is different from other HpaI enzymes such as those from *E. coli* (*Ec*HpaI) and *Sphingomonas wittichii* RW1 (*Sw*HpaI), which cannot use Zn^2+^, instead use Co^2+^, Mn^2+^, and Mg^2+^ as cofactors (27, 30, 33, 39, 40). Zn^2+^ binds to *Ab*HpaI in an octahedral coordination with six chelating atoms consisting of the pyruvate core, Glu151, Asp177, and two water molecules (W_A_ and W_S1_), so does the geometry of Co^2+^, Mn^2+^, and Mg^2+^ (Figure 3, Figure 4, Figure 5, Figure 6), which differs from Zn^2+^ tetrahedral coordination commonly found in aldolase and nonaldolase enzymes (43, 44, 45, 46). However, the Zn^2+^ octahedral coordination is similar to those found in *E. coli* RhuA, a DHAP-specific Class II metal aldolase (47). We noted an interesting cooperative enhancement of ligand (pyruvate) binding to *Ab*HpaI in the presence of Zn^2+^, Co^2+^, and Mn^2+^ (Figs. 4 and 5 and Table 1) while weaker interaction or none was observed with Mg^2+^ and Ca^2+^. This therefore suggests that the active site is more rigid upon cofactor binding, thereby enhancing the binding of pyruvate and the cleavage activities of (4*R*)-KDGal (Table 2).

Zn^2+^ cofactor also promotes the highest *R*/*S* stereoselectivity ratio in the *Ab*HpaI aldol cleavage. The preference of (4*R*)-KDGal over (4*S*)-KDGlu cleavage by *Ab*HpaI•Zn^2+^ is approximately sixfold greater than the reactions of other M^2+^ cofactors (Table 2 and Fig. 2, *A* and *B*). QM/MM MD calculations gave higher favorable binding energy of (4*R*)-KDGal leading to a more stable complex of *Ab*HpaI•Zn^2+^•(4*R*)-KDGal better poised for cleavage, explaining the stereoselectivity of *Ab*HpaI•Zn^2+^ (Table S2).

Structural analysis of *Ab*HpaI•Zn^2+^•(4*R*)-KDGal and *Ab*HpaI•Zn^2+^•(4*S*)-KDGlu complexes suggested that Arg72,Val′120, Ala′122, and Ala′123 on the pocket border from the nearby subunit (which also defines the pocket size) are key factors for stereoselectivity *via* facilitating stronger interactions with the 4-OH and D-glyceraldehyde moiety of (4*R*)-KDGal over (4*S*)-KDGlu (Fig. 4*A*). The preferred orientation of the C_4_-OH cleavage site of (4*R*)-KDGal binding to Zn^2+^ thus gives rise to a shorter distance between W_A_ water (a catalytic base) and 4-OH of (4*R*)-KDGal (3.6 Å), compared with that of (4*S*)-KDGlu (3.9 Å) (Fig. 6*A*). Comparison of the catalytic pockets between *Ab*HpaI and *Ec*HpaI (As *Ec*HpaI was solved as one protomer per asymmetric unit, thus the dimer was generated by symmetry operation) showed that the *Ec*HpaI pocket was 0.3 Å wider than that of *Ab*HapI. Moreover, HKHD bound in *Ec*HpaI (PDB code 4B5V) was found in two isomeric forms, which both interact with Arg70 and the nearby subunit residues similar to the case of *Ab*HpaI. A wider binding pocket in *Ec*HpaI may be the cause of the lacking stereoselectivity in this enzyme (23, 39). Altogether, molecular interactions between *Ab*HpaI•M^2+^ and ligand, particularly hydrogen bonding with the aldehyde moiety of substrate governed by Arg72 and coordination of the pyruvate core in the M^2+^ cofactor geometry described above, promote stereoselectivity and stereospecificity in the *Ab*HpaI•M^2+^-catalyzed aldol cleavage reaction.

*Ab*HpaI can catalyze the aldol condensation with stereospecificity and use broad aldehyde spectrum (Tables 2 and 5 and Fig. S11). Our kinetics demonstrated that *Ab*HpaI•Zn^2+^ can synthesize only (4*R*)-KDGal from pyruvate and D-glyceraldehyde. This finding suggests that *Ab*HpaI has a stereospecific control over aldol condensation reactions. The crystal structures showed that *Ab*HpaI can bind both aliphatic and aromatic aldehydes (PPA, SSA, and HBA) with a proper chemical space to satisfy van der Waals interactions between the substrate and hydrophobic residues (Trp21, Leu214, Val236, and Leu′124) (Fig. 5). Recently, aromatic substituted aldehydes have been reported in two aldolases, YfaU (a pyruvate-specific Class II metal aldolase) and 2-keto-3-deoxy-6-phosphogluconate (KDPG) aldolase (a pyruvate-specific Class I lysine aldolase); however, their crystal structures were not available (5, 25). Nonetheless, the molecular dockings were performed and showed that *N*-benzyloxycarbonyl (*N*-Cbz)-substituted aldehydes are surrounded by hydrophobic residues Trp23, Phe174, and Leu216, similar to the aldehyde binding residues identified in *Ab*HpaI. These properties offer an opportunity for *Ab*HpaI to serve as a biocatalyst to catalyze formation of 4-hydroxy-2-ketoacid and 2-keto-3-deoxy-D-sugar acid precursors valuable for the synthesis of APIs (48, 49).

**Table 5:** Products obtained from the *Ab*HpaI•Zn^2+^ catalyzed-aldol condensation of pyruvate and various aldehyde substrates

Aldehyde	Product	Product structure	Measured *m/z*
D-Glyceraldehyde	(4*R*)-KDGal		177.0401
Succinic semialdehyde	HKHD		189.0401
Propionaldehyde	4-Hydroxy-2-ketohexanoic acid		145.0543
Butyraldehyde	4-Hydroxy-2-ketoheptanoic acid		159.0664
Pentanal	4-Hydroxy-2-ketooctanoic acid		173.0825
Glutaraldehyde	7-Formyl-4-hydroxy-2-ketoheptanoic acid		187.0612
Hexanal	4-Hydroxy-2-ketononanoic acid		187.0982
Benzaldehyde	4-Hydroxy-2-keto-4-phenylbutanoic acid		193.0513
4-Hydroxybenzaldehyde	4-Hydroxy-4-(4-hydroxyphenyl)-2-ketobutanoic acid		209.0440
Anisaldehyde	4-Hydroxy-4-(4-methoxyphenyl)-2-ketobutanoic acid		223.0607

For product structures, the original pyruvate and aldehyde core structures are shown in blue and red colors, respectively. The asterisks indicate the stereocenter. The wavy bond at the stereocenter indicates the possibility to form either the *R*- or *S*-isomer.

Presently, *Ab*HpaI is the only enzyme of HpaI superfamily in the pyruvate-specific Class II metal aldolases that catalyzes stereospecific aldol condensation. While most aldolases in Class II including *Ec*HpaI, YfaU (Ni^2+^ cofactor) and putative bacterial HpaIs (Mg^2+^ cofactor) lack stereospecificity (5, 21, 22, 25). The most investigated enzyme in this superfamily, *Ec*HpaI catalyzes the condensation of pyruvate and acetaldehyde to produce two stereoisomers of 4*R*- and 4*S*-isomers of 4-hydroxy-2-oxopentanoate (HOPA) (23). The only exception previously reported to exhibit stereospecificity is *Burkholderia xenovorans* BphI aldolase (*Bx*BphI), which shares 12% amino acid sequence identity with *Ab*HpaI. *Bx*BphI can catalyze specific formation of (4*S*)-HOPA using Mn^2+^ as a cofactor (23, 32). However, the reaction of *Bx*BphI is favored toward the aldol cleavage direction. A turnover number of *Bx*BphI for aldol cleavage of (4*S*)-HOPA (4 s^−1^) is fourfold faster than the condensation (0.9 s^−1^) (24), suggesting that (4*S*)-HOPA product would be continuously cleaved during enzymatic turnovers. In contrast to *Bx*BphI, *Ab*HpaI shows a preferable aldol condensation over cleavage of ∼40-fold in *Ab*HpaI•Zn^2+^ complex (Table 3). Therefore, *Ab*HpaI can be a better candidate for stereospecific control to date.

Beyond promoting the aldol reactivity and the binding of pyruvate, Zn^2+^ also enhances the thermostability of *Ab*HpaI. The enzymatic activity of *Ab*HpaI•Zn^2+^ could be retained under very high temperature, *i.e.*, 80 °C with a half-life (*t*_*1/2*_) at ∼ 3 h (Fig. 8*A*). Moreover, *Ab*HpaI•Zn^2+^ is also stable in the presence of organic solvents such as MeOH, can, and DMSO up to 20% (v/v) (Fig. 8*B*). The data indicate that not only does Zn^2+^ serve as a catalytic cofactor, but it is also involved in the quaternary structure stabilization of *Ab*HpaI. Apart from the catalytic Zn^2+^, our structural data demonstrates that Ca^2+^ is found at the trimer center on the dimerization interface of the *Ab*HpaI hexamer, neutralizing the negative charges of three Asp51 carboxylate side chains (Fig. S9), which is unique for *Ab*HpaI and is not found in *Ec*HpaI, *Sw*HpaI, other pyruvate-specific Class II metal aldolases (23, 33, 39).

In conclusion, our results here provide insightful mechanistic and structural understanding in stereoselectivity/stereospecificity control in aldol cleavage and condensation of *Ab*HpaI. As the enzyme has broad aldehyde substrate specificity, high thermostability, and solvent tolerance, the insightful knowledge obtained from this study will serve as a basis for future rational protein engineering of *Ab*HpaI and also other HpaIs in the pyruvate-specific Class II metal aldolases to achieve the capability to synthesize tailor-made, optically pure 4-hydroxy-2-ketoacid synthons required for preparation of APIs.

## Experimental procedures

### Chemicals and reagents

All chemicals were commercially available and of analytical, high purity, and HPLC grades. Buffers used in this work were (i) buffer A: 25 mM HEPES buffer, pH 7.0 containing 100 μM PMSF and 1 mM DTT; (ii) buffer B: 25 mM HEPES buffer, pH 7.0; (iii) buffer C: 25 mM HEPES buffer, pH 7.0 containing 150 mM NaCl; (iv) buffer D: 25 mM HEPES buffer, pH 7.0 containing 400 mM NaCl; (v) buffer E: 25 mM HEPES buffer, pH 7.0 containing 15% (w/v) (NH_4_)_2_SO_4_; (vi) buffer F: 50 mM HEPES buffer, pH 7.0; (vii) buffer G: 10 mM HEPES buffer, pH 7.0 containing 150 mM NaCl; (viii) buffer H: 10 mM HEPES buffer, pH 7.0; (ix) buffer I, 0.1 M sodium acetate buffer, pH 4.6.

### Expression, purification, activity assay, and oligomeric state of AbHpaI

Recombinant *Ab*HpaI was overexpressed in *E. coli* BL21(DE3) as previously described (41). Unless otherwise indicated, purification of *Ab*HpaI was carried out at 4 °C. The cell paste (∼24 g obtained from 7.8 l culture) was resuspended in buffer A, and cells were then disrupted by ultrasonication. The broken-cell suspension was centrifuged at 36,000*g* for 40 min, and the clarified supernatant was collected as crude extract. Polyethyleneimine (PEI), at a final concentration of 0.5% (w/v), was added to the crude extract to remove nucleic acid contents. After centrifugation, the clarified supernatant was fractionated with 20 to 40% (w/v) ammonium sulfate ((NH_4_)_2_SO_4_) saturation. The protein pellet was resuspended in buffer B and dialyzed in the same buffer for 16 to 18 h. After dialysis, the dialysate was clarified by centrifugation before loading onto a DEAE-Sepharose column (172 ml, 2.5 cm × 35 cm) pre-equilibrated with buffer B. The column was washed with buffers B and C, respectively, and then eluted with a linear gradient of buffers C and D. Fractions containing *Ab*HpaI were pooled and concentrated by ultrafiltration. The enzyme solution was further purified on a Phenyl-Sepharose column (45 ml, 1.5 cm × 25 cm) pre-equilibrated with buffer E. After loading the enzyme solution, the column was washed with buffer E and then eluted with a linear gradient of buffers E and A. Fractions containing *Ab*HpaI were pooled and concentrated as described above. The concentrated enzyme solution was exchanged into buffer F using a Sephadex G-25 column. The concentration of the purified *Ab*HpaI was determined using the molar absorption coefficient of 30,035 M^−1^ cm^−1^ at absorbance 280 nm (*A*_280_), which was calculated from the deduced amino acid sequence using the online tool on the ProtParam program of ExPaSy Proteomics Server (http://web.expasy.org/protparam/). The aliquots of enzyme solution were then stored at −80 °C until used. The amount of protein was determined by Bradford assay using BSA as a protein standard. The *Ab*HpaI purity and subunit molecular weight (MW) were analyzed by 12% (w/v) SDS-PAGE.

SEC was used to determine the oligomeric state of *Ab*HpaI as described previously (50). Briefly, a Superdex 200 Increase 10/300 GL gel-filtration column equipped with an ÄKTA FPLC system (GE Healthcare) was equilibrated with buffer G at a 0.5 ml/min flow rate at 25 °C and *A*_280_ was monitored for protein elution. Protein standards with known MWs (12.4–440 kDa) were used to construct a calibration curve. The elution volume (*V*_e_) of each protein was measured, while that of blue dextran was used as the void volume (*V*_o_). The protein mass was determined from a calibration curve of the relative ratio of *V*_e_/*V*_o_ *versus* the logarithm of the protein standard MWs. The oligomeric state of *Ab*HpaI was then estimated based on the calculated subunit MW.

An LDH-coupled assay was used to determine the aldol cleavage activity of *Ab*HpaI at 25 °C using oxaloacetate (OAA) as a substrate. Briefly, the reaction contained NADH (0.2 mM), LDH (30 μg/ml), Zn^2+^ (0.5 mM), OAA (1 mM) or (4*R*)-KDGal (0.2–0.3 mM), and *Ab*HpaI (0.1 μM) in buffer F. The control reaction without enzyme was used for a background subtraction. The decrease of NADH absorbance at 340 nm can be used to infer pyruvate release from *Ab*HpaI aldol cleavage. One unit of *Ab*HpaI was defined as the amount of enzyme that consumes 1 μmol of NADH per minute.

### Measurement of metal ions in AbHpaI

The M^2+^ species in the purifed *Ab*HpaI were measured by Agilent 700 Series ICP-OES (Agilent Technologies). In total, 270 μM of the purified enzyme in buffer F was subjected to ICP-OES. The emission intensity of Zn^2+^, Mn^2+^, Co^2+^, Ni^2+^, Cu^2+^, Fe^2+^, Ca^2+^, and Mg^2+^ was detected and quantified by subtraction from the background emission intensity of a buffer blank. The concentration of each M^2+^ was determined by a calibration curve of varying concentrations (0.1–10 mg/l) of each standard M^2+^ prepared in 2% (v/v) HNO_3_ *versus* the emission intensity.

To determine the mole ratios of *Ab*HpaI and M^2+^, the apo-*Ab*HpaI was reconstituted with each of the M^2+^ ions. Briefly, the purified enzyme was treated with chelating agents, EDTA, EGTA, and Chelex 100 (Merck KGaA), to strip off the M^2+^ ions. The fivefold excess concentrations of EDTA and EGTA (5 mM) and 0.5 g of Chelex 100 were added into a 10-ml *Ab*HpaI solution (1 mM). The solution mixture was thoroughly mixed and incubated at 4 °C for 16 to 18 h to complete the metal ion chelation. The excess chelating agents and the metal ion chelation complexes were then removed by a Sephadex G-25 gel-filtration column equilibrated with buffer F to obtain apo-*Ab*HpaI. To assure that the M^2+^ ions were completely removed, the apo-*Ab*HpaI was first analyzed by ICP-OES. For the reconstitution process, each M^2+^ ion in a chloride salt form, namely ZnCl_2_, MnCl_2_, CoCl_2_, CaCl_2_, and MgCl_2_ was dissolved in the Chelex 100 treated Milli-Q Type I ultrapure water, and a fivefold excess of each M^2+^ chloride (1.5 mM) was added into a 2-ml apo-*Ab*HpaI solution (0.3 mM). All samples were mixed thoroughly and incubated at 4 °C for 16 to 18 h to reconstitute the apo-*Ab*HpaI. The excess M^2+^ in each sample was removed by a PD-10 desalting column equilibrated with buffer F to obtain a holoenzyme. The mole ratio of *Ab*HpaI and M^2+^ was then determined.

### Measurement of the K_d_ values for the AbHpaI•ligand complex

The *K*_d_ values for the binding of *Ab*HpaI with ligands including M^2+^ ions (Zn^2+^, Co^2+^, Mn^2+^, Mg^2+^, and Ca^2+^) and pyruvate were measured by MicroCal PEAQ-ITC technique (Malvern Panalytical). Briefly, a 10-ml solution of apo-*Ab*HpaI (200 μM) was dialyzed in 2 l of buffer F at 4 °C for 16 to 18 h. The dialyzed buffer was used to prepare a stock solution of each ligand. To measure the *K*_d_ value for the *Ab*HpaI•M^2+^ complex, a 200-μl solution of the apo-*Ab*HpaI (40 μM) was loaded into the sample cell and the Milli-Q Type I ultrapure water was used as a reference. Three microliters of 1.5 mM ligand solution of each M^2+^ in a syringe was continuously titrated into the sample cell (0.3 μM per each injection for 13 injections) until the ligand binding reached an equilibrium at 25 °C. The Microcal PEAQ-ITC analysis software was used to calculate the *K*_d_ using the one-site binding model. To determine the pyruvate binding constant to *Ab*HpaI, a 200-μl solution of the apo-*Ab*HpaI (40 μM) was placed in the sample cell and sequentially titrated with 3 μl of 10 mM pyruvate solution from a syringe. To determine the pyruvate binding constant to the *Ab*HpaI•M^2+^ complex, a 200-μl solution of the mixture of apo-*Ab*HpaI (40 μM) and 10 *K*_d_ of each M^2+^ was placed in the sample cell and sequentially titrated with 3 μl of a 10 mM solution mixture of pyruvate and 10 *K*_d_ of each M^2+^.

### Thermal and solvent tolerance assay

To examine the thermal tolerance of *Ab*HpaI, thermal stability and activity measurements were carried out by thermofluor and LDH-coupled assays, respectively. Thermofluor stability assays were performed as previously described (50). The *T*_*m*_ values were determined for apo-*Ab*HpaI and *Ab*HpaI•M^2+^ complexes of Zn^2+^, Co^2+^, Mn^2+^, Mg^2+^, and Ca^2+^. For thermal stability measurements of the *Ab*HpaI•Zn^2+^ complex, the purified *Ab*HpaI was first incubated at various temperatures from 25 to 85 °C for 0 to 24 h. The activity was then measured by LDH-coupled assay.

To investigate the solvent tolerance, the *T*_*m*_ values of the purified *Ab*HpaI in the presence of 0 to 50% (v/v) of polar-protic (MeOH, EtOH and IPA) and polar-aprotic (ACN and DMSO) solvents were determined as above. The percentage of relative protein stability was calculated from the *T*_*m*_ of *Ab*HpaI in the absence of solvents as 100%.

### Aldol cleavage reactions

The aldol cleavage assays were carried out by LDH-coupled assay in buffer F containing 0.2 mM NADH, 0.2 mM substrate ((4*R*)-KDGal or (4*S*)-KDGlu), 0.1 mM *Ab*HpaI, and 0.5 mM of each M^2+^ ion (Zn^2+^, Co^2+^, Mn^2+^, Mg^2+^, or Ca^2+^). The apparent rates of (4*R*)-KDGal and (4*S*)-KDGlu cleavages catalyzed by each *Ab*HpaI•M^2+^ complex were measured.

The aldol cleavage steady-state kinetics of (4*R*)-KDGal and (4*S*)-KDGlu catalyzed by *Ab*HpaI•Zn^2+^ were carried out using RapidFire high-throughput mass spectrometry. The reactions contained 0.1 mM ZnCl_2_ and the purified *Ab*HpaI in buffer H and varying concentrations of the substrate. For *K*_m_ determination, (4*R*)-KDGal (0.05–2 mM) and 5 μM *Ab*HpaI or (4*S*)-KDGlu (0.1–3.2 mM) and 40 μM *Ab*HpaI were used. Before RapidFire analysis, the reaction was quenched by an equal volume of ACN at various times (0.5–30 min) and the quenched solution was centrifuged at 12,000*g* for 10 min and filtered by a 0.22-μm nylon membrane syringe filter (FilterBio Nylon Syringe Filter) to obtain the filtrate of the remaining substrate. The substrate control reaction without the enzyme was performed. To analyze the remaining substrate, 10 μl of each filtrate was injected into the RapidFire C_18_ cartridge (G9203-80105, Agilent Technologies) with the optimized conditions set up as follows. The mobile phase reagents were 0.5% formic acid in H_2_O (A) and 100% ACN (B). The loading and washing steps were performed with 100% A at a flow rate of 1.5 and 1.25 ml/min, respectively. The elution step was carried out with isocratic solution mixture of A:B (30:70) at 0.4 ml/min flow rate. Peak areas of the remaining substrate were measured in a negative mode with a quantitative selected ion monitoring (SIM) mode to detect the *m/z* 177.0 ([M-H]^−^) of both (4*R*)-KDGal and (4*S*)-KDGlu, and the concentrations were determined from the calibration plot of substrate standard concentrations (0.025–3.2 mM) *versus* peak areas. The initial velocity (*ν*_o_) of the substrate depletion from each individual concentration of substrate was calculated from the slope of the plot between the remaining substrate and time. The plots of *ν*_o_ *versus* each substrate concentration were analyzed by Michaelis–Menten equation using the Levenberg–Marquardt algorithms in GraphPad Prism version 7 software (GraphPad Software, Inc) to determine *K*_m_ and *k*_cat_.

### Aldol condensation reactions

The aldol condensation steady-state kinetics of pyruvate and D-glyceraldehyde catalyzed by *Ab*HpaI•Zn^2+^ were monitored by the formation of (4*R*)-KDGal and (4*S*)-KDGlu using a triple-quadrupole LC/MS in a negative mode. The reactions were carried out in buffer H containing 0.1 mM ZnCl_2_, 0.5 μM purified *Ab*HpaI, and varying concentrations of the substrates. For *K*_m_ determination, varying concentrations of pyruvate (0.25–8 mM) at 30 mM D-glyceraldehyde or varying concentrations of D-glyceraldehyde (0.25–32 mM) at 4 mM pyruvate were used. The LC condition was carried out at 30 °C using a Hi-Plex H cation exchange column (8 μm, 7.7 × 300 mm) and 0.5% (v/v) formic acid in H_2_O as a mobile phase at a flow rate of 0.3 ml/min. (4*R*)-KDGal and (4*S*)-KDGlu were eluted at a retention time of 17.707 and 18.632 min, respectively. The exact *m/z* 177.0 ([M-H]^−^) of both products was monitored by a SIM mode and their concentrations were determined by using a calibration curve of each product (0.005–0.4 mM). The kinetic parameters were calculated as described above.

The aldol condensation of pyruvate and D-glyceraldehyde catalyzed by *Ab*HpaI•M^2+^ complexes of Zn^2+^, Co^2+^, Mn^2+^, Mg^2+^, and Ca^2+^ was monitored as described above. The assay reactions were carried out at 25 °C for 1 h in buffer H containing 4 mM pyruvate, 30 mM D-glyceraldehyde, 0.1 mM metal chloride, and 0.05 μM of each *Ab*HpaI•M^2+^ complex. The rate of (4*R*)-KDGal and (4*S*)-KDGlu formation was determined for each *Ab*HpaI•M^2+^ complex. Time-course synthesis of (4*R*)-KDGal was performed with 0.5 μM *Ab*HpaI•Zn^2+^ and the product was monitored for 90 h.

For analysis of a broad spectrum of aldehydes, different aldehydes PPA, SSA, butyraldehyde, pentanal, glutaraldehyde, hexanal, benzaldehyde, HBA, and anisaldehyde were used as substrates. The reactions were carried out at 25 °C for 1 h in buffer H containing 10 mM pyruvate, 5 mM aldehyde, 2 mM ZnCl_2_, and 10 μM purified *Ab*HpaI. The exact *m/z* ([M-H]^−^) of the product was monitored by high-resolution Compact QTOF (Bruker Daltonics) in negative mode, equipped with a Zorbax-eclipse C_18_ column (5 μm, 4.6 × 250 mm) ultrahigh-performance liquid chromatography (Thermo Scientific) operating at 30 °C with a flow rate of 0.5 ml/min of 0.5% (v/v) formic acid.

### Crystallization and X-ray data collection and structure determination

For crystallization, apo-*Ab*HpaI (0.7 mM) was incubated for 10 min at 25 °C in buffer F containing 52 mM pyruvate and 11 mM divalent metal chloride (ZnCl_2_ or CoCl_2_ or MnCl_2_). Crystals were grown at 18 °C in microbatch drops containing 1 μl of apo-*Ab*HpaI complex with 1 μl of buffer I containing 20 mM CaCl_2_ and 30% (v/v) 2-methyl-2,4-pentanediol (MPD) as a crystallizing agent. For SSA soaking, crystals of apo-*Ab*HpaI•M^2+^•PYR complexes were soaked in a crystallizing agent containing 18.5 mM pyruvate, 7% (v/v) glycerol, 3.6 mM divalent metal chloride (ZnCl_2_, CoCl_2_, or MnCl_2_) and 36 mM SSA in a microbatch well at 27 °C for 5 to 10 min. For PPA complex formation, crystal soaking was performed in a sitting drop well containing 25 μl of a crystallizing agent plus similar concentrations of pyruvate and ZnCl_2_, and 2.5 μl of 13.9 M PPA with 7.5 μl of 13.9 M PPA in the reservoir for 3 days at 18 °C. All soaking solutions contained 7% (v/v) glycerol for cryoprotection. Crystals of *Ab*HpaI cocomplexed with (4*R*)-KDGal and (4*S*)-KDGlu compounds and Zn^2+^/Mg^2+^ cofactors were obtained from the wells containing 1 μl crystallizing solution (40 mM CaCl_2_ and 30% (v/v) MPD in buffer I) and 1 μl of 0.18 mM *Ab*HpaI, 0.71 mM ZnCl_2_ and 50 mM (4*R*)-KDGal at 4 °C, 20 h for *Ab*HpaI•Zn^2+^•(4*R*)-KDGal, 0.35 mM *Ab*HpaI, 1.16 mM ZnCl_2_ and 66.63 mM (4*S*)-KDGlu at 4 °C, 20 h for *Ab*HpaI•Zn^2+^•(4*S*)-KDGal, and 0.37 mM *Ab*HpaI, 18.30 mM MgCl_2_ and 75 mM (4*R*)-KDGal at 4 °C, 42 h for *Ab*HpaI•Mg^2+^•(4*R*)-KDGal. A crystal of *Ab*HpaI•Zn^2+^•PYR•HBA was from a well containing 1 μl crystallizing solution (40 mM CaCl_2_, 30% (v/v) MPD and 5% (v/v) trifluoroethanol in buffer I) and 1 μl of 0.32 mM *Ab*HpaI, 1.76 mM ZnCl_2_, 5.88 mM PYR, and 73.5 mM HBA at 15 °C, 20 h. Data were collected at 100 K on a D8 venture with a microfocus TXS rotating anode and Bruker PHOTON 100 detector at the NSTDA Characterization and Testing Center (NCTC). Data processing was carried out using either PROTEUM3 software pipeline (Bruker AXS 2017) (51) or HKL-2000 (52).

Phases were calculated with Phaser (53), using *Ec*HpaI (PDB code 2V5J) (30) as a search template for molecular replacement in CCP4 suite (54). Other *Ab*HpaI structures were solved with Phaser MR using the apo-*Ab*HpaI (PDB code 7ET8) as a template. Model building and refinement were performed using Coot (55) and Refmac5 (56). The ligand dictionary was prepared using ProDrg (57). Structures were validated in Procheck (58) and the wwwPDB validation server. Data collection and refinement statistics of the *Ab*HpaI complexes were listed in Table 4. Superposition of structures was done by SSM Superposition (59). EPS was calculated by APBS-PDB2PQR tools, v2.1 (60, 61). Figures were prepared with the PyMol Molecular Graphics System, v1.8 Schrödinger, LLC. Surface area was calculated using Pisa v1.48 (62). *2mF*_obs_-*DF*_model_ maps were calculated using Refmac5 (56), and *mF*_obs_-*DF*_model_ OMIT maps with the compounds omitted were calculated using polder maps (63) in Phenix suite (64). Sequences were aligned with ClustalW v2.1 (65). The alignment was drawn with ESPript (66).

### Computational calculations

To investigate the binding interaction of *Ab*HpaI•Zn^2+^ with (4*R*)-KDGal and (4*S*)-KDGlu, QM/MM MD simulations were performed. The structures of *Ab*HpaI•Zn^2+^•(4*R*)-KDGal (PDB code 7ETC) and *Ab*HpaI•Zn^2+^•(4*S*)-KDGal (PDB code 7ETD) complexes were employed and prepared as follows. The system was truncated to a 25 Å sphere with the center on the C_4_ atom of (4*R*)-KDGal. The positions of the hydrogen atoms were located in the enzyme using the CHARMM procedure HBUILD (67). Hydrogen atoms of amino acid residues were added based on the results obtained from the PropKa (68). The atom types in the topology files were assigned according to the setup CHARMM27 parameters (69). For investigation of the enzyme–substrate interactions, the system was divided into two parts, QM and MM. The QM part consisted of substrate (either (4*R*)-KDGal or (4*S*)-KDGal), which was minimized using 1000 steps of Adopted Basis Newton-Raphson (ABNR) minimization with the AM1/CHARMM27 method. Next, the AM1/CHARMM27 MD using the leapfrog Langevin dynamics with a time step of 0.001 ps was performed at 300 K. The rest of protein, Zn^2+^, and water molecules were treated as the MM part. The system was equilibrated with QM/MM MD for 120 ps. The structures of this equilibration were collected at every 20 ps. Distances between substrate, metal ion, and the surrounding residues were determined. The binding energies of both substrates to the *Ab*HpaI•Zn^2+^ were calculated and compared.

## Data availability

Data of X-ray structures are available at Protein Data Bank under PDB codes indicated.

## Supporting information

This article contains supporting information.

## Conflict of interest

The authors declare that they have no conflicts of interest with the contents of this article.
