# Supplementary material for: Catalytic and structural insights into a stereospecific and thermostable Class II aldolase HpaI from Acinetobacter baumannii
Source: J Biol Chem. 2021 Oct 5;297(5):101280. doi: 10.1016/j.jbc.2021.101280 (PMC8560999; doi:10.1016/j.jbc.2021.101280)
Supplement: Supplemental Figures S1–S11 and Tables S1–S3 [file mmc1.pdf]

Supporting Information

Catalytic and structural insights into a stereospecific and thermostable Class II aldolase HpaI from *Acinetobacter baumannii*

Pratchaya Watthaisong[1‡], Asweena Binlaeh[1‡], Aritsara Jaruwat[2], Narin Lawan[3], Jirawat Tantipisit[4], Juthamas Jaroensuk[1], Litavadee Chuaboon[5], Jittima Phonbuppha[1], Ruchanok Tinikul[6], Pimchai Chaiyen[1], Penchit Chitnumsub[2*], Somchart Maenpuen[4*]

[1]School of Biomolecular Science and Engineering, Vidyasirimedhi Institute of Science and Technology (VISTEC), Rayong 21210, Thailand
[2]Biomolecular Analysis and Application Research Team, National Center for Genetic Engineering and Biotechnology (BIOTEC), National Science and Technology Development Agency, Pathumthani 12120, Thailand
[3]Department of Chemistry, Faculty of Science, Chiang Mai University, Chiang Mai, 50200, Thailand
[4]Department of Biochemistry, Faculty of Science, Burapha University, Chonburi 20131, Thailand
[5]School of Pharmacy, Walailak University, Nakhon Si Thammarat 80160, Thailand
[6]Department of Biochemistry and Center for Excellence in Protein and Enzyme Technology, Faculty of Science, Mahidol University, Bangkok 10400, Thailand

‡ Both authors contributed equally to this work.

List of Supplemental Materials

1. **Supplemental tables: 3**
2. **Supplemental figures and legends: 11**

Supplemental tables

Table S1
Summary of *Ab*HpaI purification steps.

Purification step	Total volume (ml)	Total protein (mg)	Total activity (unit) **	Specific activity (unit/mg)	Purification fold	Recovery yield (%)
Crude extract	119	2,407	56,721	23.6	1	100
0.5% (w/v) PEI	117	1,436	54,436	37.9	1.6	96
20-40% (w/v) $(NH_4)_2SO_4$	132	2,225	52,802	23.7	1	93
DEAE-Sepharose	46	1,019	50,943	50.0	2.1	90
Phenyl-Sepharose	10.5	678*	49,184	72.5	3	87

*The total amount of protein was obtained from 7.8 l culture.
**The total activity was determined based on the decarboxylase activity using oxaloacetate as a substrate in the *Ab*HpaI-LDH coupled assay reaction.

Table S2
Binding interaction energies of the *Ab*HpaI•Zn^{2+} complex with either (4*R*)-KDGal or (4*S*)-KDGlu at every 20 ps of equilibration for a 120 ps using QM/MM (at the AM1-CHARMM27 level) MD calculations.

Structure at equilibration time (ps)	Binding interaction energy (kcal/mol)	
	(4*R*)-KDGal	(4*S*)-KDGlu
20	−148.18	−131.85
40	−156.465	−127.815
60	−162.006	−125.812
80	−174.486	−130.539
100	−153.248	−146.012
120	−154.665	−121.42
Average ± *S.E.*	−158.175 ± 9.167	−130.575 ± 8.415

Table S3
The additional LC-ESI-QTOF-MS data for the *Ab*HpaI•Zn^{2+} catalyzed-aldol condensation of pyruvate and various aldehyde substrates.

Aldehyde	Product	Ion formula	Theoretical *m/z*	Error (mDa)	Adduct
D-Glyceraldehyde	(4*R*)-KDGal	$C_6H_9O_6$	177.0405	0.3	$[M-H]^-$
Succinic semialdehyde	HKHD	$C_7H_9O_6$	189.0404	0.3	$[M-H]^-$
Propionaldehyde	4-Hydroxy-2-ketohexanoic acid	$C_6H_9O_4$	145.0506	-3.6	$[M-H]^-$
Butyraldehyde	4-Hydroxy-2-ketoheptanoic acid	$C_7H_{11}O_4$	159.0663	-0.2	$[M-H]^-$
Pentanal	4-Hydroxy-2-ketooctanoic acid	$C_8H_{13}O_4$	173.0819	-0.6	$[M-H]^-$
Glutaraldehyde	7-Formyl-4-hydroxy-2-ketoheptanoic acid	$C_8H_{11}O_5$	187.0612	0.0	$[M-H]^-$
Hexanal	4-Hydroxy-2-ketononanoic acid	$C_9H_{15}O_4$	187.0976	-0.6	$[M-H]^-$
Benzaldehyde	4-Hydroxy-2-keto-4-phenylbutanoic acid	$C_{10}H_9O_4$	193.0506	-0.7	$[M-H]^-$
4-Hydroxybenzaldehyde	4-Hydroxy-4-(4-hydroxyphenyl)-2-ketobutanoic acid	$C_{10}H_9O_5$	209.0455	1.6	$[M-H]^-$
Anisaldehyde	4-Hydroxy-4-(4-methoxyphenyl)-2-ketobutanoic acid	$C_{11}H_{11}O_5$	223.0612	0.5	$[M-H]^-$

Supplemental figures and legends

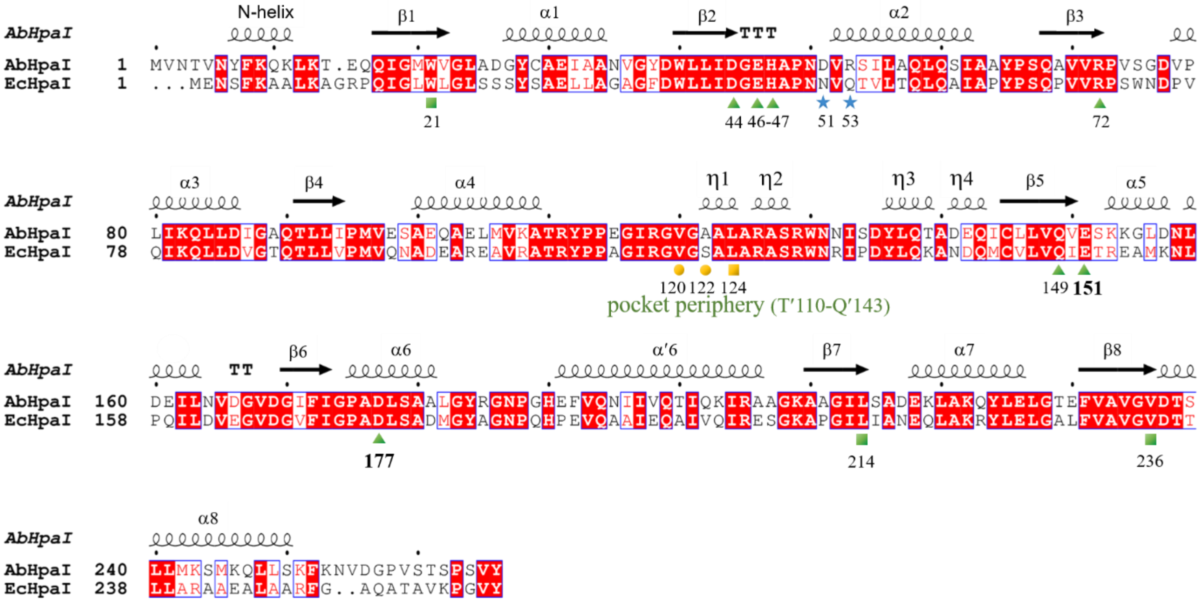

Figure S1. Amino acid sequence alignment of *Ab*HpaI and *Ec*HpaI. *Ab*HpaI shares 59% amino acid sequence identity with *Ec*HpaI. Residues in the active protomer are marked with green symbols, while the pocket periphery (T′110-Q′143) shared from the neighboring subunit of the dimer is marked with yellow symbols. Key interactions contributed from Asp44, Glu46, His47, Arg72, Glu151, Asp177, Gln149, Val′120, Ala′122 for hydrogen bonding interactions and Leu′124, Leu214 and Val236 and Trp21 for aldehyde van der Waals interactions. Residues 254-266 at the C-terminus were not observed in the structure.

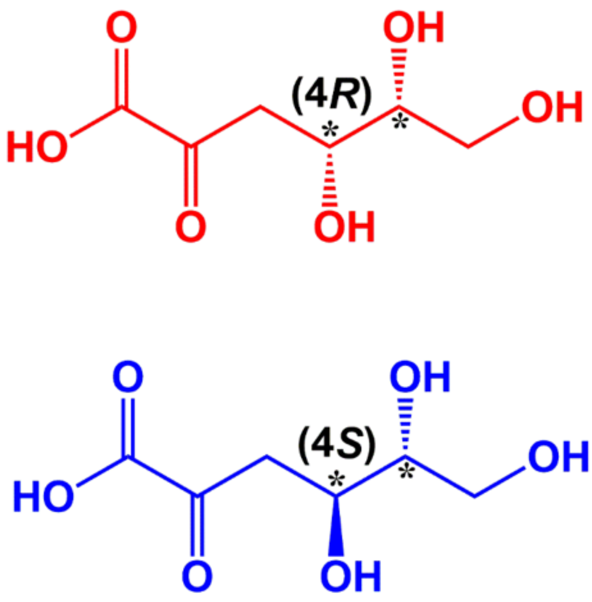

Figure S2. The chemical structures of (4*R*)-KDGal (red) and (4*S*)-KDGlu (blue). The asterisks indicate the stereocenter.

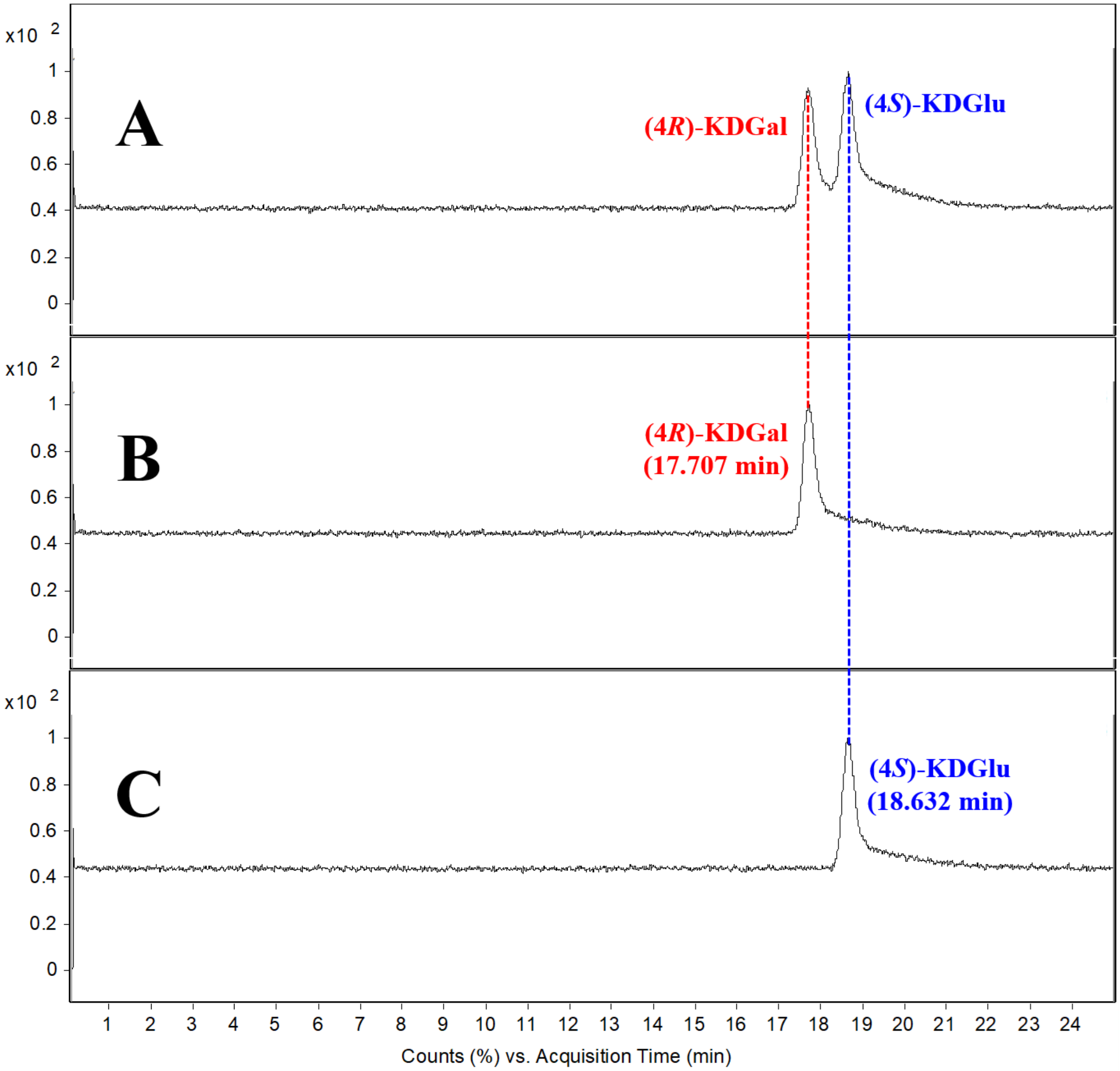

Figure S3. The separation of authentic (4*R*)-KDGal and (4*S*)-KDGlu. Panel (A) represents the total ion chromatogram (TIC) of both authentic compounds, (4*R*)-KDGal and (4*S*)-KDGlu, which are sufficiently separated by a Hi-Plex H cation-exchange column coupled with a triple quadrupole MS in negative mode, giving a retention time difference of approximately 1 min. Panels (B) and (C) indicate the TIC of (4*R*)-KDGal and (4*S*)-KDGlu at different retention times of 17.707 and 18.632 min, respectively.

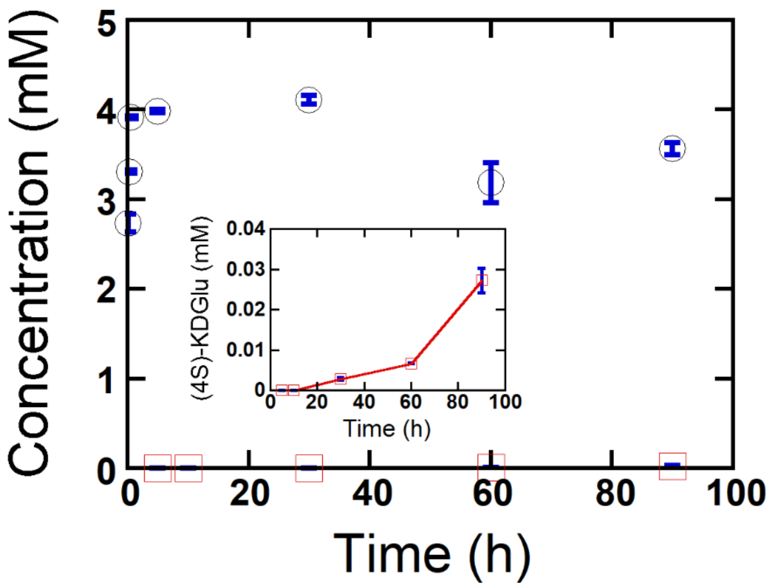

Figure S4. Time-course synthesis of (4*R*)-KDGal and (4*S*)-KDGlu. The optically pure (4*R*)-KDGal product obtained from aldol condensation of pyruvate (4 mM) and D-glyceraldehyde (30 mM) catalyzed by each *Ab*HpaI•M^{2+} (0.5 μM). The optically pure (4*R*)-KDGal product (empty circle) can be stabilized for up to 30 h in the *Ab*HpaI•Zn^{2+} (0.5 μM)-catalyzed aldol condensation of pyruvate (4 mM) and D-glyceraldehyde (30 mM) and only 0.1% of (4*S*)-KDGlu (empty square, inset) was quantitatively detected at 30 h as compared to (4*R*)-KDGal. *Error bars* represent standard deviations (S.D.) from three replications of the data.

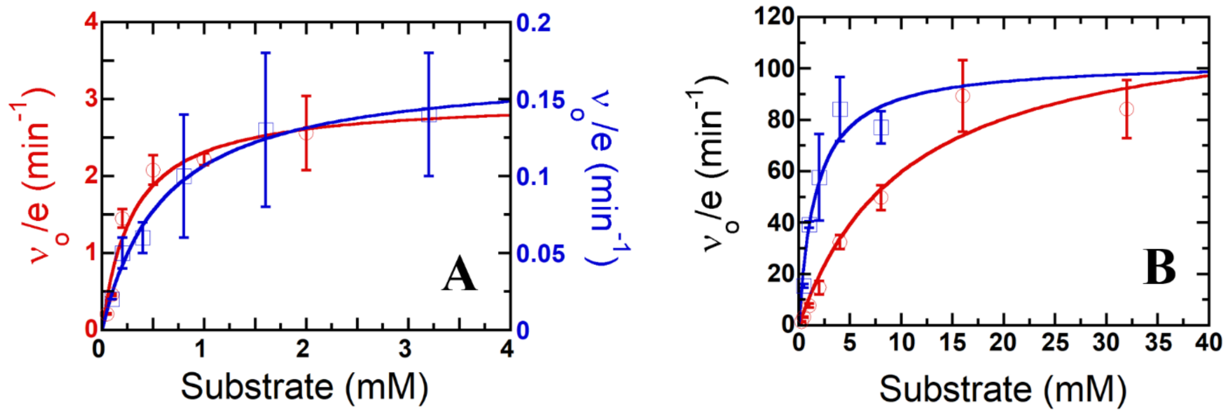

Figure S5. Michaelis-Menten plots of aldol cleavage and condensation reactions catalyzed by *Ab*HpaI•Zn^{2+}. (A) Aldol cleavage of varying substrates (4*R*)-KDGal (red line with empty circle) and (4*S*)-KDGlu (blue line with empty square) and (B) aldol condensation of varying substrates pyruvate (blue line with empty square) and D-glyceraldehyde (red line with empty circles) at a saturated concentration of another substrate. *Error bars* represent standard deviations (S.D.) from three replications of the data. The kinetic parameters are summarized in Table 3.

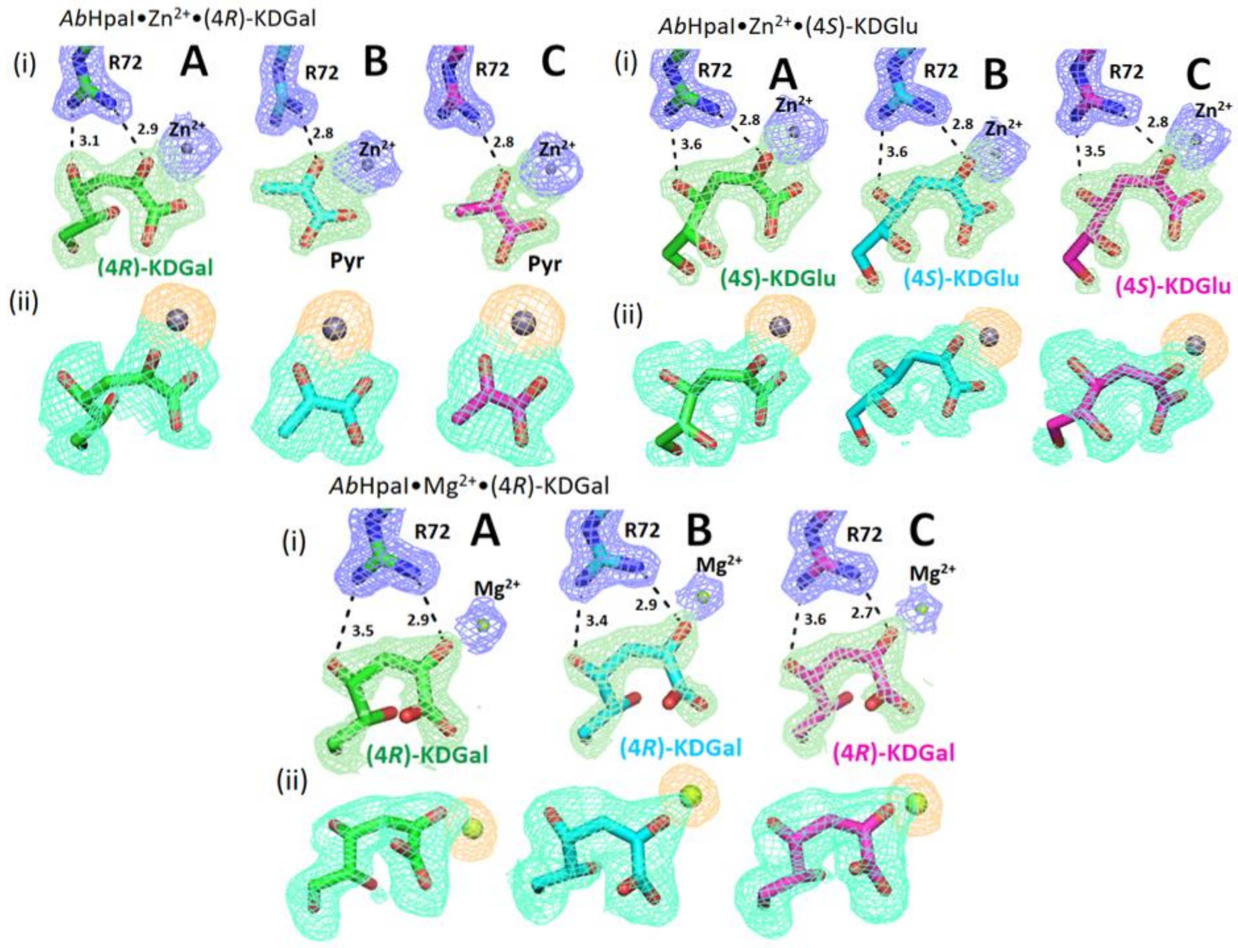

Figure S6. Density maps of (4*R*)-KDGal and (4*S*)-KDGlu substrates in *Ab*HpaI structures. (i) $2mF_{obs}$-DF_{model} maps and (ii) Polder mF_{obs}-DF_{model} OMIT maps for *Ab*HpaI•Zn^{2+}•(4*R*)-KDGal (PDB code 7ETC), *Ab*HpaI•Zn^{2+}•(4*S*)-KDGlu (PDB code 7ETD), and *Ab*HpaI•Mg^{2+}•(4*R*)-KDGal (PDB code 7ETE). The $2mF_{obs}$-DF_{model} density are contoured at 3σ for Zn^{2+}, 1.5σ for Mg^{2+} and pyruvate, 1.2σ for (4*R*)-KDGal and (4*S*)-KDGlu. Polder maps with ligands omitted are shown with mF_{obs}-DF_{model} OMIT density contoured at 3σ. Protein chains are labeled in A, B and C which represent three subunits in the asymmetric unit.

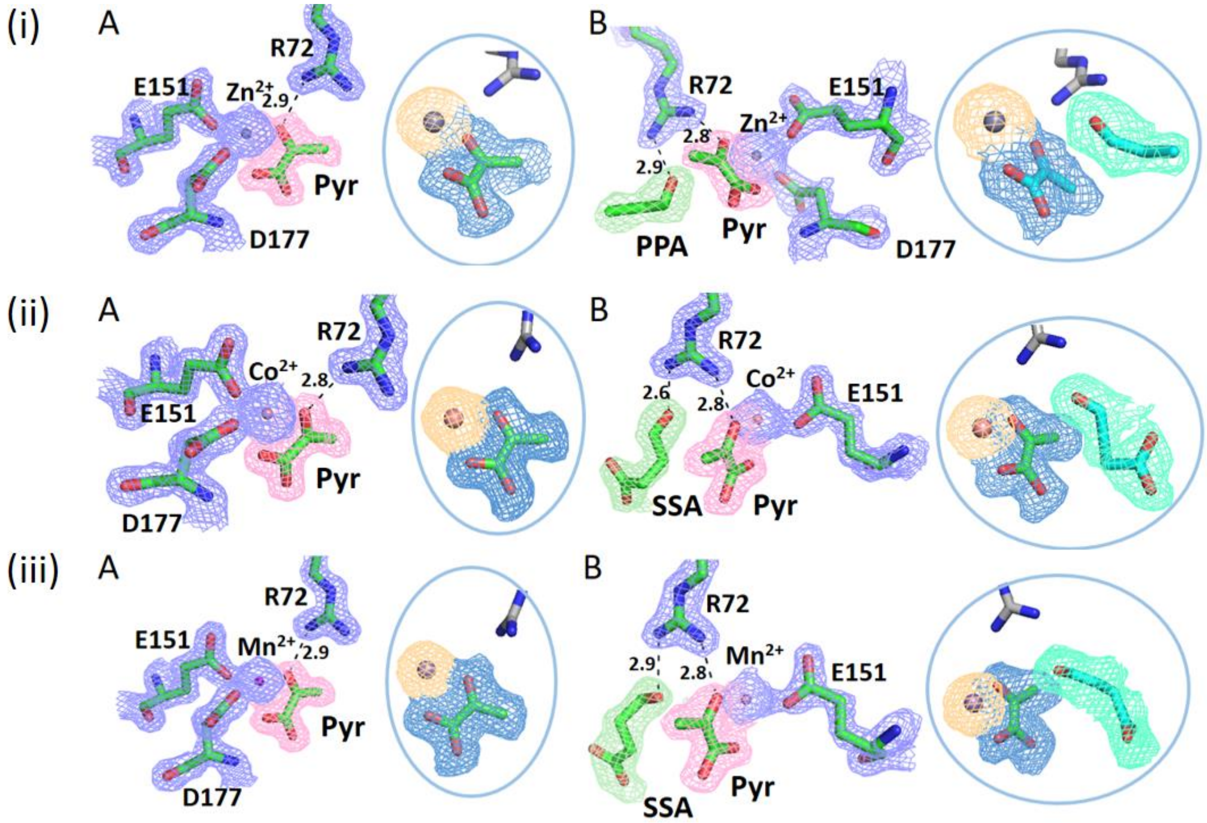

Figure S7. Density maps of pyruvate (Pyr), propionaldehyde (PPA) and succinate semialdehyde (SSA) substrates in *Ab*HpaI structures. (i) A. *Ab*HpaI•Zn^{2+}•Pyr (PDB code 7ET9), B. *Ab*HpaI•Zn^{2+}•Pyr•PPA (PDB code 7ETH), (ii) A. *Ab*HpaI•Co^{2+}•Pyr (PDB code 7ETA), B. *Ab*HpaI•Co^{2+}•Pyr•SSA (PDB code 7ETG), and (ii) A. *Ab*HpaI•Mn^{2+}•Pyr (PDB code 7ETB), B. *Ab*HpaI•Mn^{2+}•Pyr•SSA (PDB code 7ETF). The $2mF_{obs}$-DF_{model} maps are contoured at 3σ, 1.5σ and 1σ for M^{2+}, pyruvate, and PPA and SSA, respectively. Polder maps with ligand omitted are shown in a blue circle. The mF_{obs}-DF_{model} OMIT difference density are contoured at 3σ. Only one subunit is shown.

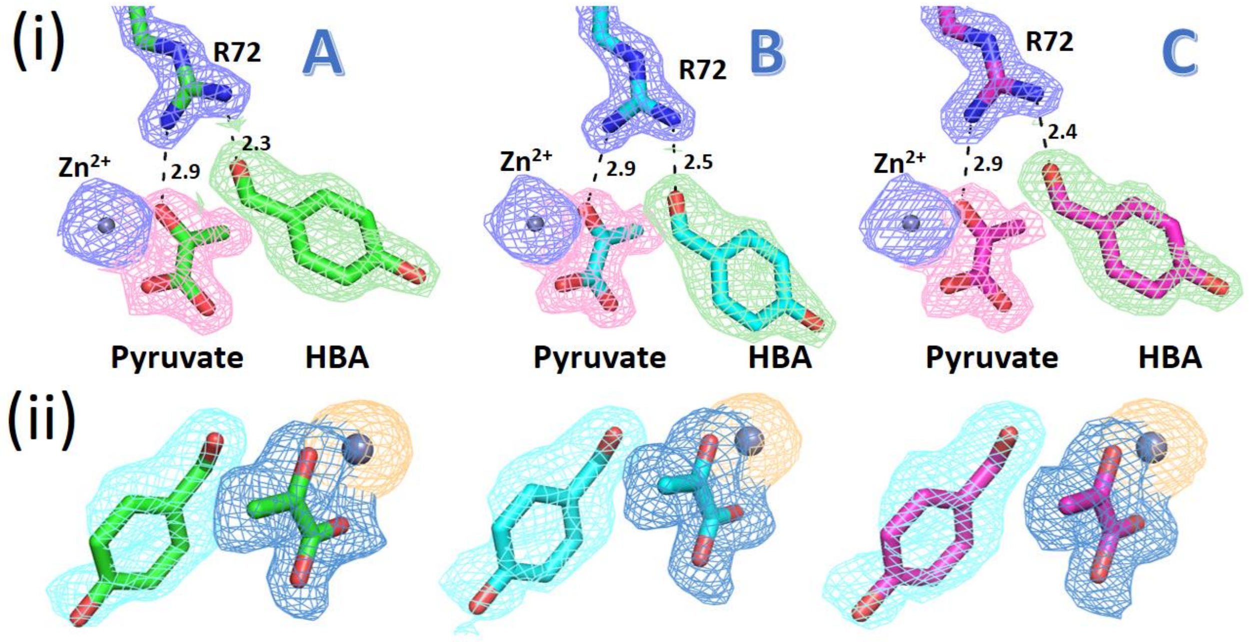

Figure S8. Density maps of pyruvate and 4-hydroxybenzaldehyde (HBA) substrates in the *Ab*HpaI structure of *Ab*HpaI•Zn^{2+}•HBA (PDB code 7ETI). (i) The $2mF_{obs}$-DF_{model} maps are contoured at 3σ, 1.5σ and 1σ for Zn^{2+}, pyruvate and HBA, respectively and (ii) Polder maps with ligands omitted are shown with mF_{obs}-DF_{model} OMIT density contoured at 3σ for ligands. Protein chains are labeled in A, B and C which represents three subunits in the asymmetric unit.

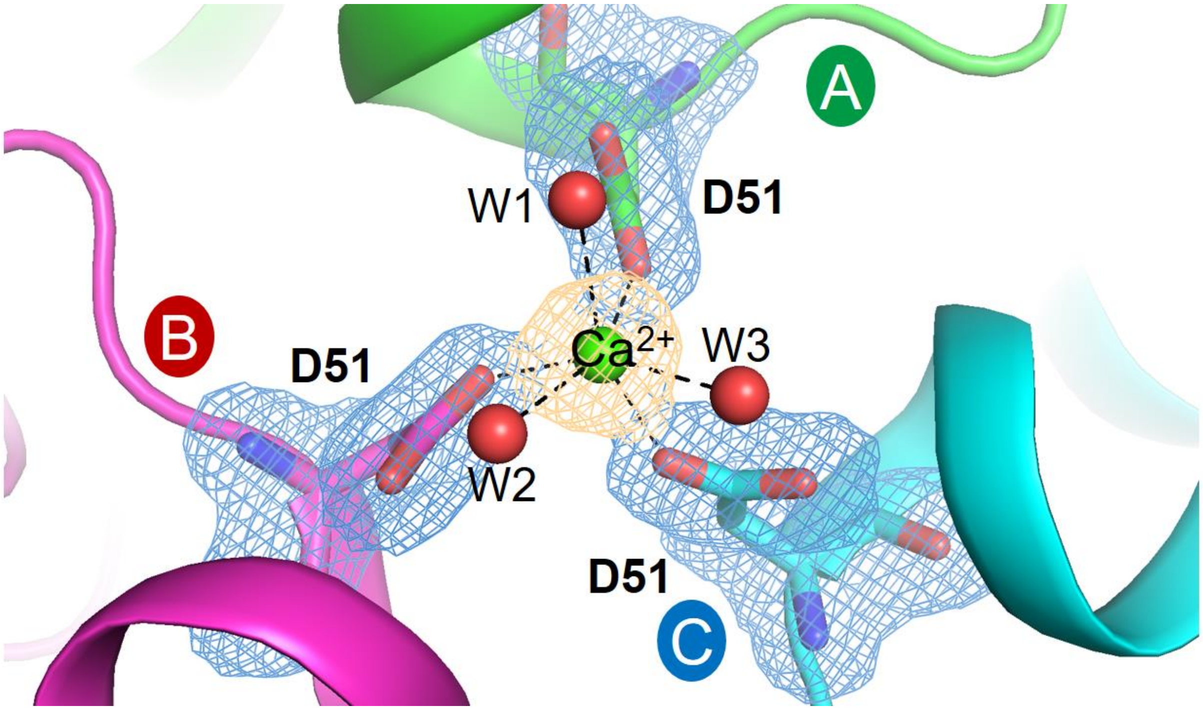

Figure S9. Omit map of Ca^{2+} ion and Asp51 at the trimer center of apo-*Ab*HpaI structure (PDB code 7ET8). Polder maps with Asp51 and Ca^{2+} ion omitted are shown with mF_{obs}-DF_{model} OMIT density contoured at 3σ. Ca^{2+} ion coordination with Asp51 from each subunit of the *Ab*HpaI trimer with three water molecules (W1-W3) in distorted octahedral geometry. Protein chains are labeled in A, B and C which represents three subunits in the asymmetric unit.

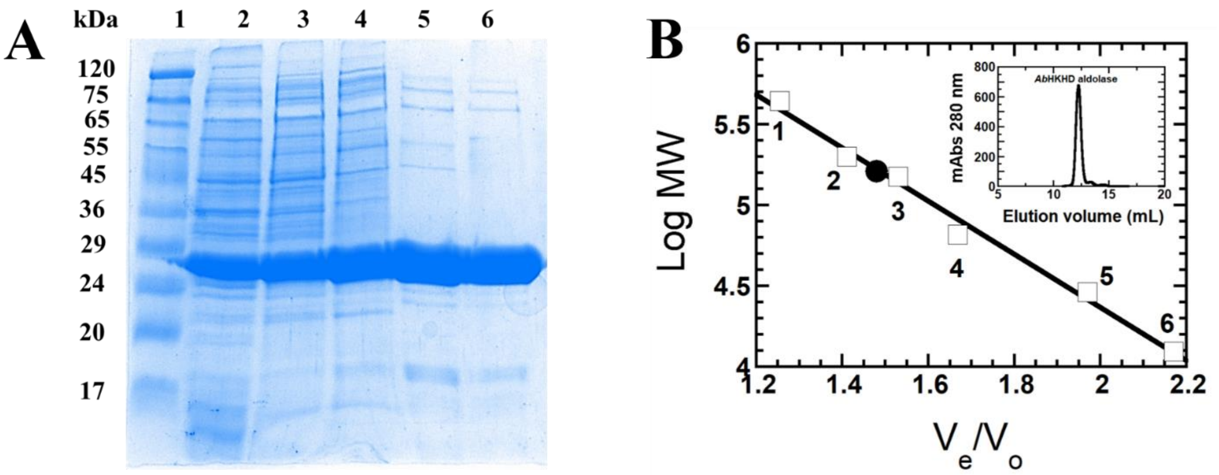

Figure S10. Purification and molecular weight (MW) of *Ab*HpaI. (A) 12% (w/v) SDS-PAGE analysis of *Ab*HpaI obtained from each purification step. Lane 1, low molecular weight protein markers (Enzmart Biotech, Thailand); lane 2, crude extract; lane 3, a supernatant obtained after precipitation by 0.5% (w/v) PEI; lane 4, after fractionation by 20-60% (w/v) $(NH_4)_2SO_4$; lane 5, after purification by DEAE-Sepharose; lane 6, after purification by Phenyl-Sepharose. A subunit MW of the purified *Ab*HpaI was estimated as approximate 29 kDa. (B) A calibration curve plot of the relative volume ratios (V_e/V_o) *versus* the logarithms of the known MW of standard proteins (□): (1) ferritin (440 kDa), (2) β-amylase (200 kDa), (3) alcohol dehydrogenase (150 kDa), (4) bovine serum albumin (66 kDa), (5) carbonic anhydrase (29 kDa) and (6) cytochrome C (12.4 kDa). *Ab*HpaI was eluted at 12.24 mL (Inset). The native MW of *Ab*HpaI was determined to be approximately 164 kDa (●). Based on the MW ratio of native to subunit form, the oligomeric state of *Ab*HpaI is hexamer.

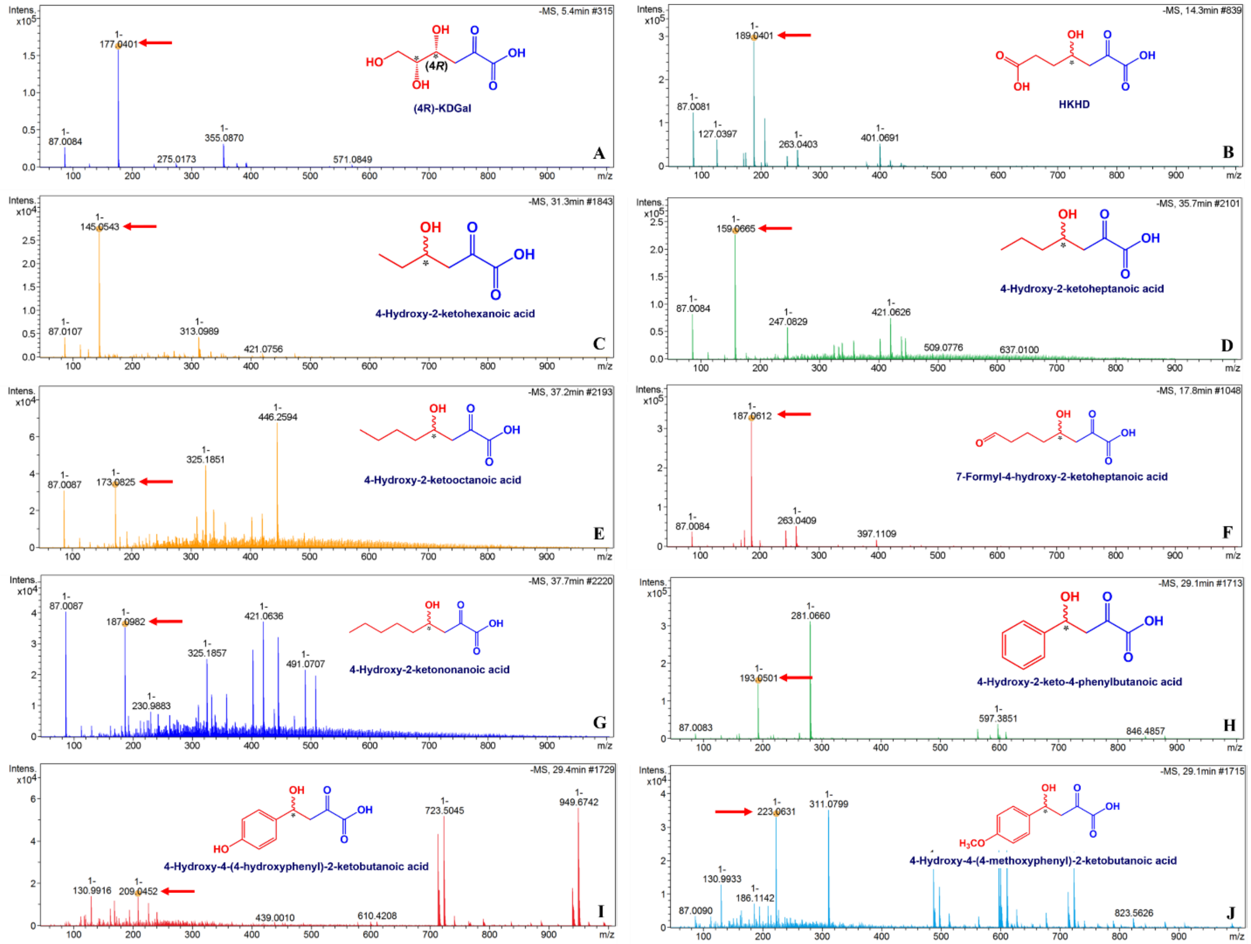

Figure S11. The exact *m/z* spectra of the aldol products obtained from the aldol condensation reaction catalyzed by *Ab*HpaI. The reactions were carried out under ambient temperature for 1 h in 10 mM HEPES buffer, pH 7.0 containing 10 µM *Ab*HpaI, 2 mM $ZnCl_2$, 10 mM pyruvate and 5 mM aldehydes (D-glyceraldehyde, SSA, propionaldehyde, butyraldehyde, pentanal, glutaraldehyde, hexanal, benzaldehyde, 4-hydroxybenzaldehyde and anisaldehyde). The control reaction for each aldol condensation reaction was also performed without addition of enzyme. The corresponding aldol products were analyzed by a high resolution ESI-QTOF-MS in negative mode. The exact *m/z* spectra of 177.0401, 189.0401, 145.0543, 159.0664, 173.0825, 187.0612, 187.0982, 193.0513, 209.0440 and 223.0607 were detected for (A) (4*R*)-KDGal, (D) HKHD, (C) 4-hydroxy-2-ketohexanoic acid, (D) 4-hydroxy-2-ketoheptanoic acid, (E) 4-hydroxy-2-ketooctanoic acid, (F) 7-formyl-4-hydroxy-2-oxoheptanoic acid, (G) 4-hydroxy-2-oxononanoic acid, (H) 4-hydroxy-2-keto-4-phenylbutanoic acid, (I) 4-hydroxy-4-(4-hydroxyphenyl)-2-ketobutanoic acid and (J) 4-hydroxy-4-(4-methoxyphenyl)-2-ketobutanoic acid, respectively.
